# Liveable residential space, residential density, and hypertension in Hong Kong: A population-based cohort study

**DOI:** 10.1371/journal.pmed.1003824

**Published:** 2021-11-02

**Authors:** Chinmoy Sarkar, Ka Yan Lai, Michael Y. Ni, Sarika Kumari, Gabriel M. Leung, Chris Webster

**Affiliations:** 1 Healthy High Density Cities Lab, HKUrbanLab, The University of Hong Kong, Hong Kong, Special Administrative Region, People’s Republic of China; 2 School of Public Health, The University of Hong Kong, Hong Kong, Special Administrative Region, People’s Republic of China; 3 The State Key Laboratory of Brain and Cognitive Sciences, The University of Hong Kong, Hong Kong, Special Administrative Region, People’s Republic of China; Harvard Medical School, UNITED STATES

## Abstract

**Background:**

Hypertension is a leading preventable risk factor of chronic disease and all-cause mortality. Housing is a fundamental social determinant of health. Yet, little is known about the impacts of liveable residential space and density on hypertension.

**Methods and findings:**

This retrospective observational study (median follow-up of 2.2 years) leveraged the FAMILY Cohort, a large territory-wide cohort in Hong Kong, Special Administrative Region, People’s Republic of China to quantify associations of objectively measured liveable space and residential density with blood pressure outcomes among adults aged ≥16 years. Blood pressure outcomes comprised diastolic blood pressure (DBP), systolic blood pressure (SBP), mean arterial pressure (MAP), and hypertension. Liveable space was measured as residential floor area, and density was assessed using the number of residential units per building block and neighborhood residential unit density within predefined catchments. Multivariable regression models examined associations of liveable floor area and residential density with prevalent and incident hypertension. We investigated effect modifications by age, sex, income, employment status, and housing type. Propensity score matching was further employed to match a subset of participants who moved to smaller residences at follow-up with equivalent controls who did not move, and generalized linear models examined the impact of moving to smaller residences upon blood pressure outcomes. Our fully adjusted models of prevalent hypertension outcomes comprised 30,439 participants at baseline, while 13,895 participants were available for incident models at follow-up. We found that each interquartile range (IQR) increment in liveable floor area was associated with lower DBP (beta [β] = −0.269 mm Hg, 95% confidence interval [CI]: −0.419 to −0.118, *p* < 0.001), SBP (β = −0.317 mm Hg, −0.551 to −0.084, *p* = 0.008), MAP (β = −0.285 mm Hg, −0.451 to −0.119 with *p* < 0.001), and prevalent hypertension (odds ratio [OR] = 0.955, 0.918 to 0.993, *p* = 0.022) at baseline. Each IQR increment in residential units per building block was associated with higher DBP (β = 0.477 mm Hg, 0.212 to 0.742, *p* = <0.001), SBP (β = 0.750 mm Hg, 0.322 to 1.177, *p* = <0.001), MAP (β = 0.568 mm Hg, 0.269 to 0.866, *p* < 0.001), and prevalent hypertension (OR = 1.091, 1.024 to 1.162, *p* = 0.007). Each IQR increase in neighborhood residential density within 0.5-mi street catchment was associated with lower DBP (β = −0.289 mm Hg, −0.441 to −0.137, *p* = <0.001), SBP (β = −0.411 mm Hg, −0.655 to −0.168, *p* < 0.001), MAP (β = −0.330 mm Hg, −0.501 to −0.159, *p* = <0.001), and lower prevalent hypertension (OR = 0.933, 0.899 to 0.969, *p* < 0.001). In the longitudinal analyses, each IQR increment in liveable floor area was associated with lower DBP (β = −0.237 mm Hg, −0.431 to −0.043, *p* = 0.016), MAP (β = −0.244 mm Hg, −0.444 to −0.043, *p* = 0.017), and incident hypertension (adjusted OR = 0.909, 0.836 to 0.988, *p* = 0.025). The inverse associations between larger liveable area and blood pressure outcomes were more pronounced among women and those residing in public housing. In the propensity-matched analysis, participants moving to residences of lower liveable floor area were associated with higher odds of incident hypertension in reference to those who did not move (OR = 1.623, 1.173 to 2.199, *p* = 0.002). The major limitations of the study are unmeasured residual confounding and loss to follow-up.

**Conclusions:**

We disentangled the association of micro-, meso-, and macrolevel residential densities with hypertension and found that higher liveable floor area and neighborhood scale residential density were associated with lower odds of hypertension. These findings suggest adequate housing in the form of provisioning of sufficient liveable space and optimizing residential density at the building block, and neighborhood levels should be investigated as a potential population-wide preventive strategy for lowering hypertension and associated chronic diseases.

## Abstract 2

In a cohort study, Dr. Chinmoy Sarkar and colleagues investigate the association between liveable residential space, residential density and hypertension in Hong Kong.

## Author summary

**Why was this study done?:**

- Hypertension is the leading preventable risk factor of cardiovascular disease (CVD) and all-cause mortality.
- The fundamental role of housing in shaping population health has been acknowledged by the World Health Organization (WHO) and the American Heart Association.
- However, scientific evidence with regard to the relationship of housing overcrowding and residential density with hypertension has been insufficient.

**What did the researchers do and find?:**

- We examined associations of objectively measured liveable space and multiscalar residential density with hypertension among adults employing 2 consecutive waves of a large territory-wide cohort in Hong Kong, Special Administrative Region, People’s Republic of China.
- At baseline, each interquartile range (IQR) increment in liveable floor area was associated with lower blood pressure and prevalent hypertension. At the block level, each IQR increase in residential units per building block was associated with higher blood pressure and prevalent hypertension. At a neighborhood level, higher residential density was associated with lower blood pressure and hypertension.
- Our longitudinal analysis found that each IQR increment in liveable floor area was associated with 9.1% lower odds of incident hypertension.
- Propensity-matched analysis found that in reference to participants who did not move, those relocating to residences of lower liveable floor area was associated with higher odds of hypertension.

**What do these findings mean?:**

- Provisioning adequate liveable space and optimizing density of residential units at building block and neighborhood levels may act as population-wide preventive strategies for lowering the burden of hypertension.
- The evidence can inform housing policy toward health-centric housing space allocation and densification while planning, designing, and retrofitting high-density cities of similar contexts.

## Introduction

A third of all adults are affected by hypertension, representing the leading preventable risk factor of cardiovascular disease (CVD) and all-cause mortality globally [1,2]. A study of hypertension in 154 countries projected that an estimated 874 million adults had systolic blood pressure (SBP) ≥140 mm Hg, resulting in 7.8 million deaths and 143 million disability-adjusted life years (DALYs) [3]. In Hong Kong, Special Administrative Region, People’s Republic of China, 31.6% of the population aged 15 years and above have hypertension, and only half of the patients with hypertension (46.2%) were aware of their condition [4]. The public health benefits of lowering of blood pressure and hypertension prevention, especially with respect to reducing the burden of cardiovascular and other chronic diseases, have been well documented [5,6]. Recently, the Lancet Commission on Hypertension has also highlighted the role of health-promoting environments in prevention of hypertension [7]. Few studies have thus far examined the link between built environment exposures and hypertension, reporting negative associations with walking behavior [8,9], neighborhood walkability [10,11], and green exposures [12,13].

Housing constitutes the predominant component of built environment in cities and a key social determinant of health [14–17]. Its importance has been exemplified by the recent World Health Organization (WHO) guidelines [18], as well as the AHA Scientific Statement on housing and health [19]. Among housing characteristics, inadequate liveable space constitutes one of the primary determinants of health, being a marker of the degree of overcrowding and associated chronic environmental stress [20,21]. Previous evidence on the adverse health effects of density or crowdedness have emerged from animal experimental studies. In one of the seminal animal studies, John Calhoun, an American ethologist found that rodents confined to environments of increasing density, albeit with provisions of sufficient necessities including food and water reported pathological behaviors including elevated mortality, reduced fertility rates, social withdrawal, and reproductive disorders among female [22]. Other animal studies have corroborated similar negative health impacts [23–26]. Evidence from human studies have been scarce and far from conclusive. One of the first human studies had found that the number of persons per room and housing units per structure were the 2 primary determinants of social pathologies [27]. A few cross-sectional ecological studies have reported detrimental effects of overcrowding on blood pressure levels [28,29].

At a community level, residential density measured in terms of number of housing units within a neighborhood constitutes a fundamental metric defining allocation of housing stocks and associated services and facilities. Residential density has been established as a fundamental proxy for liveable and accessible communities [30]. Higher neighborhood-level residential density supports compact, mixed-use walkable communities and is a requisite for efficient public transport systems. It has been found to be beneficially associated with active travel, physical activity, lower adiposity, nonsedentary behaviors, and lower private vehicle miles [31–33]. The evidenced density effects have been attributed to compact walkable environments that promote adequate levels of physical activity.

Nonetheless, most prior studies on housing and health have been at an ecological scale. Furthermore, to the best of our knowledge, there has been no prior longitudinal evidence linking objective measures of housing environments measured at individual level and cardiovascular risk in high-density environments. The present study aims to investigate independent associations of objectively measured housing environment (liveable area and density) with blood pressure outcomes and risk of hypertension in the highly dense environmental setting of Hong Kong, Special Administrative Region, People’s Republic of China. We also aim to explore effect modifications by age, sex, income, employment status, and housing type and the potential impacts of moving to residences of smaller liveable area upon hypertension.

## Methods

In this retrospective cohort study, we employed data from the baseline and second waves of the Hong Kong FAMILY Cohort to examine the longitudinal associations of housing environment with blood pressure outcomes and hypertension. The FAMILY Cohort is a population-based prospective cohort study of physical, mental, and social well-being. The baseline phase recruited 46,001 participants from 20,279 households achieving a response rate of 21.7%. This constituted just under 1% of Hong Kong’s total 2,368,796 households. Participants were recruited from 6 sources. These included (1) population representative stratified random sampling of households across all districts of Hong Kong; (2) sampling of first-degree relatives of the randomly sampled participants; (3) additional sampling of residents of 3 relatively remote new towns and districts of Tung Chung, Tin Shui Wai, and Tseung Kwan O and (4) purposive sampling of special groups of interest such as households with newly married couples; (5) households with a child entering first year of primary school; and (6) with at least 1 hospitalized family member. The family residing in the same household constituted the unit of sampling. In the second wave of household visit (wave 2), 32,016 completed the survey with 2,837 newly recruited members, with a follow-up rate of 69.6%. Detailed data on sociodemographics, lifestyle, behaviors, anthropometrics, blood pressure, happiness, and family harmony were collected across both the waves [4].

For the present study, we identified target samples of 37,656 participants at baseline and 25,209 at wave 2 after excluding participants aged <16 years and with missing outcomes and exposure variables (Figs B and C in S3 Appendix).

The study was approved by the Institutional Review Board of the University of Hong Kong/Hospital Authority Hong Kong West Cluster. Written informed consent was obtained from all the participants of the study.

### Outcomes

Diastolic blood pressure (DBP) and SBP in mm Hg were measured by trained assessors with the help of Omron HEM-7000 electronic blood pressure monitor and expressed as the mean of 2 readings taken at least 5 minutes apart. Mean arterial pressure (MAP) was calculated by dividing the pulse pressure (measured as the difference between SBP and DBP) by 3 and adding this value to the DBP, to represent the steady blood pressure component and act as a marker of left ventricular contractility, heart rate, and peripheral vascular resistance. A case of hypertension was defined as mean DBP ≥ 90 mm Hg, mean SBP ≥ 140 mm Hg, and/or self-reported current antihypertensive medication usage as per the 2018 ESC/ESH guidelines [34]. Data on antihypertensive medication usage was assessed from a questionnaire on self-reported usage of doctor-prescribed Western medicine in the past 6 months (see Table A in S2 Appendix for specification), either solely or in conjunction with other medications. Additionally, as secondary outcomes, we also measured the change in blood pressure outcomes from baseline phase to wave 2 in terms of increments in DBP, SBP, and MAP by ≥10% relative to the baseline value. Participants with missing data across 2 consecutive blood pressure readings or on antihypertension medication usage at baseline or wave 2 were excluded from analysis.

### Exposures

We developed the Hong Kong Housing Environment Database (HKHED), a geospatial database of multiscalar measures of residential density within building blocks and neighborhoods; liveable floor areas; and levels in permanent residential units of public housing, subsidized housing and private housing including village houses and villas (illustrated in Fig A in S3 Appendix). The GIS database of individual building footprints, housing estate boundaries, and street centreline (IG1000) was sourced from the Hong Kong Lands Department, while details of housing units within individual blocks were collected from multiple government and private sources including The Hong Kong Housing Authority (HA), Hong Kong Housing Society (HS), Hong Kong Government’s GeoInfo Map, Home Affairs Department, and Centaline Property Agency (CentaMap). The dataset employed to measure participants’ neighborhood residential density comprised 2.53 and 2.58 million housing units at the end of the baseline and the follow-up waves, respectively, which correspond well with the housing figures published by the Transport and Housing Bureau [35].

The FAMILY Cohort participants’ dwelling addresses were first geocoded at the building block level and subsequently matched with the exact floor level housing data within the HKHED. We were able to geocode 19,719 (97.2%) and 14,113 (93.1%) of the residential addresses at baseline and wave 2, respectively. At a microlevel, residential floor area in square feet was employed as an objective measure of liveable space. The data were sourced from HA, HS, and CentaMap and linked to geocoded participants’ addresses. At a mesolevel, residential density of a participant’s building block was expressed as the total number of residential units within the block where the participant resided. It is a function of building height, building footprint, and design. At the macrolevel of neighborhood, residential density expressed as units per km^2^ was measured within behaviorally relevant neighborhoods. Street catchments of 0.5 (803 m) and 1 mile (1,609 m) of the participants’ geocoded dwelling addresses, equivalent to a 10- and 15-minute walk from the dwellings, respectively, were employed as functional neighborhoods. Hong Kong residents walk an average of 10 to 15 minutes to access public transport [36,37]. Our models also adjusted for floor level of residence extracted from the participants’ anonymized residential address data. The floor level data were further corrected in building blocks that skipped certain levels for auspicious reasons in the Hong Kong context. Corrections were made to the participant’s floor level if they resided in building blocks with floor levels ending with “4” (i.e., 4, 14, and 24), starting with “4” (i.e., 40 to 49), and/or having numbers with inauspicious meaning (e.g., 24 in Cantonese has a phonetic pronunciation as “easy death” and 58 as “not prosper”). In total, 5.2% and 5.6% of the floor level at baseline and the first follow-up were adjusted upon rigorous checking, mostly in private residential blocks built beyond mid-1990s.

Among the other neighborhood environment variables, public transport density was defined as the number of transport stations including bus, green minibus, mass transit railway (MTR), light rail, trams, and ferries within street catchments of the participants’ residences. The primary public transport data were obtained from various government sources (https://data.gov.hk/en-data/dataset/hk-td-tis_3-routes-and-fares-of-public-transport) including the Lands Department iB10000 GIS layer. To account for terrain-related walking impedance, we measured terrain variability, expressed in terms of standard deviation (SD) in slope (in degrees), employing a 5-meter digital terrain model (https://data.gov.hk/en-data/dataset/hk-landsd-openmap-5m-grid-dtm) within neighborhood catchments of 0.5 and 1 mile.

The housing and the neighborhood environment exposures were assessed at the end of each wave (baseline and wave 2). Participants who changed residences across the 2 waves were identified, and their new housing environment exposures were measured. All spatial analyses were conducted in geographic information system software ArcGIS version 10.6.

### Statistical analysis

We excluded all participants aged below 16 years. Model covariates and putative confounders for adjustment were selected a priori based on published literature and assumed causal relationships [34,38]. Participants’ age in years was calculated from the dates of birth to examination dates at baseline and wave 2 (coded as ≤40, >40 to ≤50, >50 to ≤60, >60). Other sociodemographic covariates comprised sex (male versus female), marital status (coded as 3-factor variable: never married, married, and widowed/divorced/separated), highest educational qualification (primary, secondary, and tertiary or higher degrees), employment status (employed, homemaker/student/others, and retiree/unemployed), and personal income including individual social security payments (4 factor variable as <HK$5,000, 5,000 to 9,999, 10,000 to 14,999, ≥15,000; HK$1.0 = US$0.13). Lifestyle level variables comprised smoking status (binary nonsmoker/past smoker and current smoker), alcohol intake frequency (coded as nondrinker/former drinker, occasional to 1 to 3 per month and 1 to 3 per week up to daily), and number in the family (5-factor variable: 1, 2, 3, 4, and ≥5), shared living (coded as single-family, 2-family, 3-family, and >3-family households), and house type (binary public housing, private housing or subsidized sale flats). Comorbidities derived from medical history comprised doctor-diagnosed coronary heart disease and obesity/overweight coded as a binary variable. Among the housing environment exposure variables, liveable floor area, residential units per building block and neighborhood residential density were expressed in terms of per-interquartile increment. Floor level was coded as a 4-factor variable (0 to 5, 6 to 10, 11 to 20, and >20). Other neighborhood environment variables adjusted for in our models included density of public transport (expressed as per interquartile range [IQR] increment) and terrain variability (1 SD in slope). Time of follow-up adjusted in longitudinal models of incident hypertension was measured from examination date at baseline to wave 2 (expressed as logarithm of follow-up years). A series of linear and logistic regression models was fitted to examine the associations of blood pressure outcomes (DBP, SBP, and MAP) and prevalent hypertension, respectively, with residential environment measured within 0.5-mile residential catchment at baseline and wave 2. Model building comprised sequentially introducing blocks of sociodemographic, lifestyle, and comorbidity variables, examining multicollinearity and model fit at each step to ensure parsimony. Longitudinal analyses comprised continuous outcome models examining associations of DBP, SBP, and MAP with residential environment adjusting for time of follow-up and restricted to participants who were free from hypertension at baseline. Logistic regression models further examined the associations between incident hypertension among nonhypertensive participants from baseline. Minimally (age and sex) adjusted and fully adjusted models were presented.

We further conducted a series of sensitivity analyses to test for the robustness of our reported results. These comprised (1) repeating analyses with multiple imputation for missing data using the multiple imputation by chained equations (MICE) in Stata [39]. Firstly, to account for missingness across the covariates, our imputation models for target samples (37,656 at baseline, 25,209 at wave 2, and 16,388 for longitudinal analyses) included all covariates, and exposure and outcome variables used in the fully adjusted complete case analyses, and imputed missing data for marital status, personal income, education, employment status, smoking status, alcohol frequency, doctor-diagnosed coronary heart disease, obesity/overweight, and housing type. Secondly, as a further sensitivity test, we additionally imputed missing data on account of loss to follow-up in our imputation models of 37,656 participants employing data available on outcomes, exposures, and covariates at baseline and wave 2. A total of 20 imputation sets were created [40,41]; (2) examining the fully adjusted associations, with neighborhood-level built environment measured at a spatial scale of 1-mile (1,609 m) residential street catchment (i.e., extending the size of the neighborhood); (3) We reran longitudinal models with secondary outcomes of ≥10% increase in DBP, SBP, and MAP over the follow-up period. (4) As a further sensitivity test, we reran our models using household income instead of personal income. (5) We also repeated analyses rerunning models with age taken as a continuous variable. (6) We performed stratified analyses to examine effect modifications by subgroups of age, sex, income, employment status, and housing type categories on the associations of floor area and residential units per building block upon blood pressure outcomes. (7) Lastly, we examined the association of decrement in liveable floor area with incident hypertension outcomes as a further test to support underlying mechanism. We performed propensity score matching in a subsample of participants who did not change home address (controls) and those who moved to a dwelling of reduced liveable floor area (treatment group). Participants were first assigned to control and treatment groups based on residential moves over the follow-up period. The liveable floor area distribution of controls was further restricted on the basis of liveable floor area distribution of the treatment group at follow-up. Subsequently propensity score models were developed, reflecting the propensity of moving from a bigger to smaller residence, adjusting all covariates and confounding variables before the move (age, sex, marital status, personal income, employment status, doctor-diagnosed coronary heart disease, obesity/overweight, number of family members, smoking status, and hypertension and floor area at baseline). The control group was matched with the treatment group at a 1:1 ratio employing the nearest neighbor method. Finally, generalized linear models were fitted examining the odds of incident hypertension, ≥10% increment in blood pressure as well as blood pressure outcomes at follow-up in the treatment groups in reference to the control, adjusting for propensity score [42]. As a further sensitivity test, we reran the generalized linear models adjusting for all baseline covariates and confounding variables employed to derive the propensity score.

We report effect estimates: beta (β) for continuous models and odds ratios (ORs) for binary models and 2-tailed 95% confidence intervals (CIs) estimated with robust variance estimator (Huber–White sandwich estimator) to account for potential clustering within data. All analyses were conducted in statistical packages Stata 16. R package *MatchIt* was employed for propensity score estimation and matching [43].

Although all analyses were prespecified, our study did not have a specific prospective analysis plan. Changes to the planned analysis made in compliance with reviewer’s suggestions included adjustments for shared living and housing type, subgroup level analyses by employment status and housing type, and additional sensitivity tests with age as a continuous variable. Post hoc corrections were made to the floor level data. Post hoc secondary analysis included models adjusting for household income instead of personal income and liveable floor area expressed as per 100 square feet increment. The study is reported as per the Strengthening the Reporting of Observational Studies in Epidemiology (STROBE) guideline (S1 Appendix).

## Results

After excluding participants aged <16 years and with missing outcomes and exposure variables, the target sample at baseline with complete data on outcomes and exposures comprised 37,656 participants recruited between February 28, 2009 and March 30, 2011, of which 14,495 (38.5%) were lost to follow-up. The target sample for wave 2 contained 25,209 participants recruited over August 3, 2011 and June 19, 2013, of which 1,418 were newly recruited participants. We assumed missingness to be at random and that the missing data were associated with observed data [40]. To further examine missingness due to exclusions, we first conducted Little’s test for missing completely at random (MCAR) using *mcartest* command in Stata; however, our results did not support the MCAR assumption. As an additional test to decide between MCAR and missing at random (MAR), we performed independent samples *t* test on each covariate by the missingness indicators for age, outcomes, and exposure variables (basis of exclusions), and obtained significant results (*p* < 0.05) implying the excluded participants can be treated as MAR and not MCAR. The summary characteristics of the longitudinal sample are presented in Table 1. At baseline, the mean age of the sample was 47.0 years (SD = 17.6), and 54.5% were female. Prevalent cases of hypertension constituted 26.8% and 21.2% in baseline and wave 2, respectively. The mean liveable floor area was 458.4 (SD = 160.1) at baseline and 454.3 square feet (SD = 157.4) at wave 2. The mean residential units per building block at baseline and follow-up were 470.4 (SD = 302.2) and 471.5 (301.7), while the mean neighborhood-level residential density within a 0.5-mi neighborhood catchment were 28,850.0 (SD = 11,225.9) and 29,407.7 units/km^2^ (SD = 11,122.6), respectively.

**Table 1:** Descriptive characteristics of the longitudinal sample.

Participant characteristics	Baseline (*N* = 37,656)	Wave 2 (*N* = 22,330^a^)	Effect size^b^^,^^c^
*Covariates*			
Age in years (mean, SD)	47.0 (17.6)	49.6 (17.2)	0.15
Sex *N* (%): Female	20,537 (54.5)	12,330 (55.2)	0.01
Male	17,119 (45.5)	10,000 (44.8)	
Highest educational qualification *N* (%): Primary	9,709 (25.8)	5,827 (26.1)	0.03
Secondary	17,971 (47.7)	10,098 (45.2)	
Tertiary	9,794 (26.0)	6,373 (28.5)	
Missing	182 (0.5)	32 (0.1)	
Marital status *N* (%): Never married	9,270 (24.6)	4,584 (20.5)	0.05
Married	24,830 (65.9)	15,202 (68.1)	
Widowed/divorced/separated	3,502 (9.3)	2,514 (11.3)	
Missing	54 (0.1)	30 (0.1)	
Employment status *N* (%): Employed	18,186 (48.3)	10,863 (48.7)	0.03
Home maker/student/others	10,681 (28.4)	5,851 (26.2)	
Retiree/unemployed	5,140 (13.7)	3,279 (14.7)	
Missing	3,649 (9.7)	2,337 (10.5)	
Personal income (HK$; HK$ 1.0 = US$0.13): <4,999	16,046 (42.6)	8,873 (39.7)	0.06
5,000 to 9,999	6,749 (17.9)	3,896 (17.5)	
10,000 to 14,999	5,316 (14.1)	3,481 (15.6)	
≥15,000	6,735 (17.9)	5,083 (22.8)	
Missing	2,810 (7.5)	997 (4.5)	
Smoking status *N* (%): Nonsmoker/past smoker	32,198 (85.5)	19,664 (88.1)	0.04
Current smoker	5,418 (14.4)	2,634 (11.8)	
Missing	40 (0.1)	32 (0.1)	
Alcohol consumption *N* (%): Never/former drinker	28,481 (75.6)	17,259 (77.3)	0.03
Occasional/1 to 3 per month	6,200 (16.5)	3,694 (16.5)	
1 to 3 per week to daily	2,734 (7.3)	1,331 (6.0)	
Missing	241 (0.6)	46 (0.2)	
Number of family members *N* (%): living alone	5,422 (14.4)	3,062 (13.7)	0.04
2	12,072 (32.1)	8,021 (35.9)	
3	9,311 (24.7)	5,441 (24.4)	
4	7,723 (20.5)	4,120 (18.5)	
≥5	3,128 (8.3)	1,686 (7.6)	
Number of core family *N* (%): Single family	22,579 (60.0)	13,608 (60.9)	0.04
2 families	6,099 (16.2)	3,905 (17.5)	
3 families	3,860 (10.3)	2,342 (10.5)	
>3 families	5,118 (13.6)	2,475 (11.1)	
Overweight or obese *N* (%): BMI<23	17,528 (46.6)	9,705 (43.5)	0.03
BMI ≥23	19,422 (51.6)	11,944 (53.5)	
Missing	706 (1.9)	681 (3.1)	
Self-reported coronary heart disease *N* (%): No	36,346 (96.5)	21,614 (96.8)	<0.01
Yes	1,054 (2.8)	593 (2.7)	
Missing	256 (0.7)	123 (0.6)	
*Outcomes*			
DBP, mm Hg (mean, SD)	78.7 (11.4)	76.3 (10.9)	0.22
SBP, mm Hg (mean, SD)	125.9 (20.5)	121.9 (19.1)	0.20
MAP, mm Hg (mean, SD)	94.4 (13.5)	91.5 (12.7)	0.22
*Built environment variables*			
Floor area, square feet (mean, SD)	458.4 (160.1)	454.3 (157.4)	0.03
Housing units per block (mean, SD)	470.4 (302.2)	471.5 (301.7)	<0.01
Residential unit density within 0.5 mile (units/km^2^) (mean, SD)	28,850.0 (11,225.9)	29,407.7 (11,122.6)	0.05
Residential unit density within 1 mile (units/km^2^) (mean, SD)	21,381.5 (10,128.3)	21,635.6 (8,768.1)	0.03
Housing type *N* (%): Public housing	17,856 (47.4)	10,544 (47.2)	<0.01
Nonpublic housing	19,799 (52.6)	11,786 (52.8)	
Missing	1 (0.0)	0 (0.0)	
Floor level (mean, SD)	15.8 (10.4)	16.1 (10.7)	0.02
Public transport density within 0.5 mile (units/km^2^) (mean, SD)	41.2 (21.3)	40.8 (21.1)	0.02
Public transport density within 1 mile (units/km^2^) (mean, SD)	35.7 (17.7)	35.4 (17.5)	0.01
Terrain variability within 0.5 mile (SD, degrees) (mean, SD)	10.7 (4.8)	10.8 (4.9)	0.02
Terrain variability within 1 mile (SD, degrees) (mean, SD)	11.8 (4.2)	11.8 (4.2)	0.01

^a^ Included only participants who were followed up across baseline and the first follow-up (i.e., new recruits at wave 2 were not included).

^b^ Cohen’s *w* effect size for categorical variable: 0.1, small; 0.3, medium; and 0.5, large.

^c^ Cohen’s *d* effect size for continuous variable: 0.2, small; 0.5, medium; and 0.8, large.

BMI, body mass index; DBP, diastolic blood pressure; km, kilometer; MAP, mean arterial pressure; SBP, systolic blood pressure; SD, standard deviation.

### Cross-sectional association of liveable area and housing density with prevalent hypertension

Cross-sectional complete case analyses at baseline and wave 2 comprised analytic samples of 30,439 and 20,244 participants, respectively (see Fig B in S3 Appendix for sample selection and Table B in S2 Appendix for summary characteristics). Models of associations of housing environment with blood pressure outcomes and prevalent hypertension in both waves are presented in Tables 2 and 3 (for full models, see Table C in S2 Appendix). Each IQR increment in liveable floor area (183.0 square feet) was significantly associated with lower blood pressure outcomes (β_DBP_ = −0.269 mm Hg, 95% CI: −0.419 to −0.118, *p* < 0.001; β_SBP_ = −0.317 mm Hg, −0.551 to −0.084, *p* = 0.008; β_MAP_ = −0.285 mm Hg, −0.451 to −0.119, *p* < 0.001) and lower prevalent hypertension (adjusted OR = 0.955, 0.918 to 0.993, *p* = 0.022). The results in wave 2 also remained consistent, with each IQR increment being associated with lower prevalent hypertension (adjusted OR = 0.932, 0.884 to 0.983, *p* = 0.009). At the building block level, each IQR increment in housing units in a block (490 units/block) was significantly associated with higher blood pressure outcomes (β_DBP_ = 0.477 mm Hg, 0.212 to 0.742; β_SBP_ = 0.750 mm Hg, 0.322 to 1.177; β_MAP_ = 0.568 mm Hg, 0.269 to 0.866 with *p* < 0.001) and higher prevalent hypertension (adjusted OR = 1.091, 1.024 to 1.162, *p* = 0.007) at baseline. Each IQR increase in neighborhood-level residential density (13,931 units/km^2^) measured within 0.5-mi street catchment was associated with lower blood pressure (β_DBP_ = −0.289 mm Hg, −0.441 to −0.137; β_SBP_ = −0.411 mm Hg, −0.655 to −0.168; β_MAP_ = −0.330 mm Hg, −0.501 to −0.159 with *p* < 0.001) and lower odds of hypertension (adjusted OR = 0.933, 0.899 to 0.969, *p* < 0.001). The variance inflation factor of all our models ranged between 1.06 and 1.87, indicating low multicollinearity.

**Table 2:** Cross-sectional association of housing environment exposures with measures of blood pressure outcomes and prevalent hypertension among FAMILY Cohort participants aged 16 or above at baseline.

Housing environment^a^	DBP (mm Hg)	SBP (mm Hg)	MAP (mm Hg)	Hypertension
	β (95% CI) *p*-value	β (95% CI) *p*-value	β (95% CI) *p*-value	OR (95% CI) *p*-value
**Model 1**^**b**^, *n* = 37,656				
Floor area, square feet (per IQR)	−0.602 (−0.735, −0.469) <0.001	−1.124 (−1.333, −0.916) <0.001	−0.776 (−0.924, −0.628) <0.001	0.882 (0.853, 0.911) <0.001
Housing units per block (per IQR)	0.601 (0.408, 0.795) <0.001	0.828 (0.516, 1.140) <0.001	0.677 (0.459, 0.894) <0.001	1.086 (1.040, 1.133) <0.001
Neighborhood residential density, units/km^2^ (0.5 mi, per IQR)	−0.180 (−0.317, −0.043) 0.010	−0.198 (−0.417, 0.020) 0.075	−0.186 (−0.340, −0.032) 0.018	0.971 (0.941, 1.002) 0.065
**Model 2**^**c**^, *N* = 30,439				
Floor area, square feet (per IQR)	−0.269 (−0.419, −0.118) <0.001	−0.317 (−0.551, −0.084) 0.008	−0.285 (−0.451, −0.119) <0.001	0.955 (0.918, 0.993) 0.022
Housing units per block (per IQR)	0.477 (0.212, 0.742) <0.001	0.750 (0.322, 1.177) <0.001	0.568 (0.269, 0.866) <0.001	1.091 (1.024, 1.162) 0.007
Neighborhood residential density, units/km^2^ (0.5 mi, per IQR)	−0.289 (−0.441, −0.137) <0.001	−0.411 (−0.655, −0.168) <0.001	−0.330 (−0.501, −0.159) <0.001	0.933 (0.899, 0.969) <0.001

^a^ Model with neighbourhood environment (residential density, density of public transport and terrain) measured within 0.5-mile (805m) street catchment of geocoded participants’ residence.

^b^ Models adjusting for age and sex.

^c^ Fully-adjusted models accounting for socio-demographics (age, sex, marital status, employment status, educational attainment, income), lifestyle (smoking status, alcohol intake frequency, number of family members, shared living, housing type), comorbidities (obesity, cardiac heart disease) and environment (housing floor level, density of public transport and terrain).

β, beta; CI, confidence interval; DBP, diastolic blood pressure; IQR, interquartile range; km, kilometer; MAP, mean arterial pressure; mi, mile; OR, odds ratio; SBP, systolic blood pressure.

**Table 3:** Cross-sectional association of housing environment exposures with measures of blood pressure outcomes and prevalent hypertension among FAMILY Cohort participants aged 16 or above in the first follow-up (wave 2).

Housing environment^a^	DBP (mm Hg)	SBP (mm Hg)	MAP (mm Hg)	Hypertension
	β (95% CI) *p*-value	β (95% CI) *p*-value	β (95% CI) *p*-value	OR (95% CI) *p*-value
**Model 1**^**b**^, *n* = 25,209				
Floor area, square feet (per IQR)	−0.530 (−0.688, −0.371) <0.001	−1.013 (−1.264, −0.763) <0.001	−0.691 (−0.865, −0.517) <0.001	0.864 (0.827, 0.903) <0.001
Housing units per block (per IQR)	0.612 (0.388, 0.836) <0.001	0.768 (0.411, 1.126) <0.001	0.664 (0.415, 0.913) <0.001	1.051 (0.995, 1.110) 0.072
Neighborhood residential density, units/km^2^ (0.5 mi, per IQR)	−0.047 (−0.210, 0.116) 0.569	−0.142 (−0.397, 0.114) 0.278	−0.079 (−0.260, 0.102) 0.394	1.024 (0.983, 1.067) 0.252
**Model 2**^**c**^, *N* = 20,244				
Floor area, square feet (per IQR)	−0.320 (−0.496, −0.144) <0.001	−0.433 (−0.706, −0.160) 0.002	−0.358 (−0.547, −0.169) <0.001	0.932 (0.884, 0.983) 0.009
Housing units per block (per IQR)	0.419 (0.114, 0.724) 0.007	0.603 (0.114, 1.092) 0.016	0.480 (0.140, 0.820) 0.006	1.068 (0.984, 1.159) 0.118
Neighborhood residential density, units/km^2^ (0.5 mi, per IQR)	0.044 (−0.134, 0.222) 0.628	−0.088 (−0.371, 0.195) 0.543	0.0001 (−0.199, 0.199) 0.999	1.016 (0.968, 1.068) 0.518

^a^ Model with neighborhood environment (residential density and density of public transport and terrain) measured within 0.5-mile (805 m) street catchment of geocoded participants’ residence.

^b^ Models adjusting for age and sex.

^c^ Fully adjusted models accounting for sociodemographics (age, sex, marital status, employment status, educational attainment, and income), lifestyle (smoking status, alcohol intake frequency, number of family members, shared living, and housing type), comorbidities (obesity and cardiac heart disease), and environment (housing floor level and density of public transport and terrain).

β, beta; CI, confidence interval; DBP, diastolic blood pressure; IQR, interquartile range; km, kilometer; MAP, mean arterial pressure; mi, mile; OR, odds ratio; SBP, systolic blood pressure.

### Longitudinal association of liveable area and housing density with incident hypertension

In our longitudinal analyses of incident hypertension, we linked cohort data across baseline and second waves. Of the target sample of 25,209 participants in wave 2, after excluding those who were newly recruited in wave 2, had missing outcomes or exposure data at baseline, were diagnosed with hypertension at baseline and had missing data on covariates at wave 2, an analytic sample of 13,895 participants with observations for 30,162.7 person-years at risk was used (see Fig C in S3 Appendix). A total of 1,333 new hypertension cases (9.6%) were identified over the median follow-up period of 2.2 years (range: 1.3 to 3.6 years). After excluding participants who were hypertensive at baseline and further adjusting for age, sex, and follow-up time, we found that liveable floor area was inversely associated with blood pressure outcomes and hypertension, while the associations were opposite for building block density. In fully adjusted models (see Table 4), each IQR increment in floor area was associated with lower blood pressure outcomes (β_DBP_ = −0.237 mm Hg, −0.431 to −0.043, *p* = 0.016 and β_MAP_ = −0.244 mm Hg, −0.444 to −0.043, *p* = 0.017) and incident hypertension (adjusted OR = 0.909, 0.836 to 0.988, *p* = 0.025). This is equivalent to 5.1% lower odds of hypertension for every 100 square feet increment in floor area (adjusted OR = 0.949, 0.907 to 0.993, *p* = 0.025). Table D in S2 Appendix presents models in terms of 100 square feet increments in floor area. As previously, at the building block level, higher block density was associated with higher blood pressure outcomes (β_DBP_ = 0.338 mm Hg, 0.011 to 0.666, *p* = 0.043; β_SBP_ = 0.589 mm Hg, 0.097 to 1.082, *p* = 0.019; and β_MAP_ = 0.422 mm Hg, 0.070 to 0.774, *p* = 0.019).

**Table 4:** Longitudinal association of housing environment exposures with blood pressure outcomes and incident hypertension among FAMILY Cohort participants aged 16 or above who were followed up and had not been diagnosed as hypertensives at baseline.

Housing environment^a^	DBP (mm Hg)	SBP (mm Hg)	MAP (mm Hg)	Incident hypertension*
	β (95% CI) *p*-value	β (95% CI) *p*-value	β (95% CI) *p*-value	OR (95% CI) *p*-value
**Model 1**^b^, *n* = 16,388				
Floor area, square feet (per IQR)	−0.361 (−0.538, −0.185) <0.001	−0.544 (−0.799, −0.290) <0.001	−0.422 (−0.609, −0.236) <0.001	0.876 (0.816, 0.940) <0.001
Housing units per block (per IQR)	0.396 (0.148, 0.645) 0.002	0.697 (0.328, 1.065) <0.001	0.496 (0.229, 0.764) <0.001	1.017 (0.931, 1.112) 0.703
Neighborhood residential density, units/km^2^ (0.5 mi, per IQR)	0.055 (−0.121, 0.230) 0.541	−0.130 (−0.392, 0.132) 0.331	−0.007 (−0.197, 0.183) 0.944	0.998 (0.933, 1.067) 0.946
**Model 2**^c^, *n* = 13,895				
Floor area, square feet (per IQR)	−0.237 (−0.431, −0.043) 0.016	−0.256 (−0.528, 0.016) 0.065	−0.244 (−0.444, −0.043) 0.017	0.909 (0.836, 0.988) 0.025
Housing units per block (per IQR)	0.338 (0.011, 0.666) 0.043	0.589 (0.097, 1.082) 0.019	0.422 (0.070, 0.774) 0.019	1.030 (0.905, 1.172) 0.657
Neighborhood residential density, units/km^2^ (0.5 mi, per IQR)	0.073 (−0.117, 0.263) 0.450	−0.136 (−0.425, 0.152) 0.355	0.003 (−0.203, 0.210) 0.975	0.981 (0.906, 1.062) 0.634

^a^ Model with neighborhood environment (residential density and density of public transport and terrain) measured within 0.5-mile (805 m) street catchment of geocoded participants’ residence.

^b^ Models adjusting for age at baseline, sex, and logarithm of follow-up time.

^c^ Fully adjusted models accounting for sociodemographics (age at baseline, sex, marital status, employment status, educational attainment, and income), lifestyle (smoking status, alcohol intake frequency, number of family members, shared living, and housing type), comorbidities (obesity and cardiac heart disease), environment (housing floor level and density of public transport and terrain), and logarithm of follow-up time.

^*^ The number of hypertension cases in the fully adjusted model was 1,333, and cumulative incidence was 9.6%.

β, beta; CI, confidence interval; DBP, diastolic blood pressure; IQR, interquartile range; km, kilometer; MAP, mean arterial pressure; mi, mile; OR, odds ratio; SBP, systolic blood pressure.

### Sensitivity analyses

**Fig 1:**
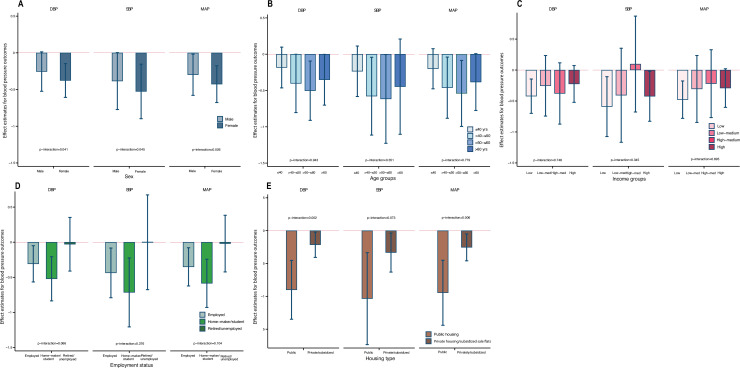
**Association between liveable floor area and blood pressure outcomes by (A) sex, (B) age groups, (C) income categories, (D) employment status, and (E) housing type in the second wave of FAMILY Cohort.** Models adjusted for sociodemographics (age, sex, marital status, employment status, educational attainment, and income), lifestyle (smoking status, alcohol intake frequency, number of family members, shared living, and housing type), comorbidities (obesity and cardiac heart disease), and environment (residential units per block, neighborhood-level residential density, housing floor level, and density of public transport and terrain). The vertical bars indicate the effect estimate (β), while the whiskers indicate 95% CI. The *p*-values are for the interactions between liveable floor area and population subgroups stratified by sex, age, income, employment status, and housing type. β, beta; CI, confidence interval; DBP, diastolic blood pressure; MAP, mean arterial pressure; SBP, systolic blood pressure.

**Fig 2:**
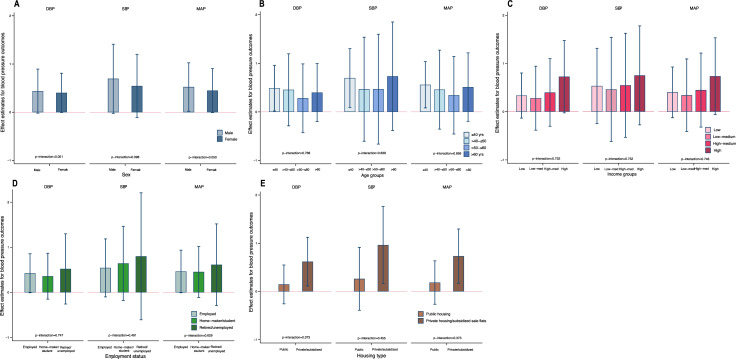
**Association between residential units per block and blood pressure outcomes by (A) sex, (B) age groups, (C) income categories, (D) employment status, and (E) housing type in the second wave of FAMILY Cohort.** Models adjusted for sociodemographics (age, sex, marital status, employment status, educational attainment, and income), lifestyle (smoking status, alcohol intake frequency, number of family members, shared living, and housing type), comorbidities (obesity and cardiac heart disease), and environment (liveable floor area, neighborhood-level residential density, housing floor level, and density of public transport and terrain). The vertical bars indicate the effect estimate (β), while the whiskers indicate 95% CI. The *p*-values are for the interactions between residential units per block and population subgroups stratified by sex, age, income, employment status, and housing type. β, beta; CI, confidence interval; DBP, diastolic blood pressure; MAP, mean arterial pressure; SBP, systolic blood pressure.

Our imputed models accounting for missingness across covariates produced results with similar effect sizes and *p*-values as with our main findings. The results also remained consistent in our models imputing both missing covariates as well as loss to follow-up (see Tables E and F in S2 Appendix). Rerunning our prevalent and incident models with housing and neighborhood environment measured at 1-mile (1,609 m) residential street catchment produced consistent results (see Tables G–I in S2 Appendix). Rerunning our longitudinal models for at least 10% increments in SBP, DBP, and MAP in the follow-up produced consistent negative associations with liveable floor area (see Table 5); however, our fully adjusted models remained significant only in the case of liveable floor area for models of ≥10% increase MAP (OR_≥10%MAP_ = 0.939, 0.882 to 0.998, *p* = 0.044). Repeating our analyses with household income instead of personal income as an additional sensitivity test produced results consistent with our primary analysis (Table J in S2 Appendix). Similarly repeating analysis with age as a continuous variable produced consistent results (Table K in S2 Appendix). We further tested for effect modification (by sex, age, income, employment status, and housing type) in the associations of liveable floor area and building unit per block with SBP, DBP, and MAP among the participants at the second wave (see Figs 1 and 2 and Table L in S2 Appendix). The results of our interaction effects models show that the negative associations of higher liveable floor area with blood pressure outcomes were significantly more pronounced in females (p_interaction_ = 0.041 for DBP, 0.045 for SBP, and 0.026 for MAP). Also, the associations were more pronounced among participants living in public housing as compared to private or subsidized sale flats (p_interaction_ = 0.002 for DBP and 0.006 for MAP). The positive associations between residential units per building block and blood pressure outcomes were also more pronounced among male participants for MAP (p_interaction_ = 0.050).

**Table 5:** Longitudinal association of housing environment exposures with 10% increase in blood pressure outcomes (DBP, SBP, and MAP) among FAMILY Cohort participants aged 16 or above who were followed up and had not been diagnosed as hypertensives at baseline.

Housing environment^a^	DBP increase of ≥10% OR (95% CI) *p*-value	SBP increase of ≥10% OR (95% CI) *p*-value	MAP increase of ≥10% OR (95% CI) *p*-value
Number of cases*	2,608	2,172	2,158
Cumulative incidence (%)*	18.8	15.6	15.5
**Model 1**^**b**^, *n* = 16,388			
Floor area, square feet (per IQR)	0.936 (0.889, 0.986) 0.013	0.941 (0.891, 0.994) 0.030	0.927 (0.879, 0.979) 0.006
Housing units per block (per IQR)	1.046 (0.976, 1.121) 0.204	1.013 (0.941, 1.090) 0.737	1.004 (0.932, 1.080) 0.925
Neighborhood residential density, units/km^2^ (0.5 mi, per IQR)	0.978 (0.931, 1.028) 0.388	0.966 (0.915, 1.019) 0.205	0.974 (0.923, 1.028) 0.336
**Model 2**^**c**^, *n* = 13,895			
Floor area, square feet (per IQR)	0.945 (0.890, 1.004) 0.065	0.960 (0.900, 1.023) 0.206	0.939 (0.882, 0.998) 0.044
Housing units per block (per IQR)	0.993 (0.901, 1.095) 0.890	0.928 (0.835, 1.032) 0.167	0.925 (0.833, 1.027) 0.144
Neighborhood residential density, units/km^2^ (0.5 mi, per IQR)	0.970 (0.917, 1.026) 0.292	0.964 (0.908, 1.024) 0.237	0.961 (0.904, 1.022) 0.202

^a^ Model with neighborhood environment (residential density, density of public transport and terrain) measured within 0.5-mile (805m) street catchment of geocoded participants’ residence.

^b^ Models adjusting for age at baseline, sex and logarithm of follow-up time.

^c^ Fully adjusted models accounting for sociodemographics (age at baseline, sex, marital status, employment status, educational attainment, and income), lifestyle (smoking status, alcohol intake frequency, number of family members, shared living, and housing type), comorbidities (obesity and cardiac heart disease), environment (housing floor level and density of public transport and terrain), and logarithm of follow-up time.

^*^For the fully adjusted models only.

β, beta; CI, confidence interval; DBP, diastolic blood pressure; IQR, interquartile range; km, kilometer; MAP, mean arterial pressure; mi, mile; OR, odds ratio; SBP, systolic blood pressure.

Table 6 shows the ORs for incident hypertension and blood pressure outcomes for participants moving from higher to a lower liveable floor area residence in reference to propensity score matched controls. Our treatment groups (movers to lower liveable space) and controls (nonmovers) were adequately matched (see Fig D in S3 Appendix). Participants moving from higher to lower liveable floor area residence had consistently higher odds of incident hypertension (OR = 1.623, 1.173 to 2.199, *p* = 0.002).

**Table 6:** Effect of moving to residences of lower liveable area (in reference to propensity-matched nonmovers) upon incident hypertension and blood pressure outcomes at wave 2.

	Propensity score–matched cases (number of cases)	Model 1 β (95% CI) *p*-value	Model 2 β (95% CI) *p*-value
DBP*	748	1.124 (0.144, 2.104) 0.025	1.109 (0.190, 2.028) 0.018
SBP*	748	1.092 (−0.463, 2.647) 0.169	1.057 (−0.265, 2.379) 0.117
MAP*	748	1.114 (0.027, 2.200) 0.045	1.092 (0.115, 2.068) 0.028
		OR (95% CI) *p*-value	OR (95% CI) *p*-value
Incident hypertension*	748 (72)	1.623 (1.173, 2.199) 0.002	1.818 (1.292, 2.508) <0.001
DBP increase of ≥10%*	748 (147)	1.233 (0.949, 1.586) 0.109	1.251 (0.961, 1.608) 0.088
SBP increase of ≥10%*	748 (114)	1.210 (0.905, 1.593) 0.185	1.234 (0.920, 1.629) 0.148
MAP increase of ≥10%*	748 (123)	1.184 (0.884, 1.560) 0.244	1.196 (0.891, 1.578) 0.220

Model 1 is a generalized linear model examining the effects in the treatment group (moving from higher livable area to lower) in reference to the control (participants not moving residence) adjusting for propensity score. Model 2 adjusted for age, sex, marital status, personal income, employment status, coronary heart disease, number of family members, overweight or obese, smoking status, and floor area at baseline.

* All hypertension cases at baseline were excluded.

β, beta; CI, confidence interval; DBP, diastolic blood pressure; MAP, mean arterial pressure; OR, odds ratio; SBP, systolic blood pressure.

## Discussion

In a large population-based cohort study conducted in the extreme high-density setting of Hong Kong, we found that each IQR increment in liveable floor area (183 square feet) was associated with lower blood pressure outcomes and hypertension, the results being consistent in both prevalent and incident models after adjusting for sociodemographic and lifestyle factors. At a building block level, each IQR increment in block density (490 units/block) was associated with higher blood pressure outcomes and hypertension in our prevalent models, while an IQR increase in neighborhood-level density measured within 0.5-mile residential catchment was associated with blood pressure outcomes and hypertension, but only in our baseline prevalent models. Our findings remained consistent across all the blood pressure and hypertension outcomes and in models with built environment measured at a scale of 1-mile residential catchment.

At a microscale, more liveable floor area was found to be independently and consistently associated with lower blood pressure levels and odds of hypertension in both prevalent and incident models. Overall, we report 9.1% lower odds of incident hypertension per IQR increment (or 5.1% lower odds of hypertension per 100 square feet increment in liveable floor area). Furthermore, at the mesoscale of building block level, each IQR increment in residential units was consistently associated with higher SBP, DBP, and MAP in our cross-sectional and longitudinal analyses. In our analyses, lower levels of private liveable area and higher levels of living units in a building block acted as proxies of overcrowding and density. The exact mechanism linking them to elevated blood pressure has been understudied, and further research needs to focus on the underlying pathways. Relatedly, a series of animal studies linking exposure to crowded environments with social pathologies has been reported [22,25,26]. One of the first studies conducted in Chicago aiming to replicate Calhoun’s mouse model work in a human context found that the number of persons per room employed as a surrogate of crowding was the most important metric correlated with the social pathologies of mortality ratio, general fertility rate, ineffectual parental care, and juvenile delinquency, while housing units per building structure was the second most crucial determinant of social deviations [27]. It has been suggested that household overcrowding may induce a loss of privacy and resulting in a surfeit of stimuli. The resulting unwanted social contact and the inability to segregate oneself in a limited space environment may produce psychosocial stress resulting in elevated blood pressure and other chronic diseases [19,20,44]. It has been posited that stress-related psychosocial factors may result in sympathetic activation leading to the pathogenesis of hypertension [45–47]. Among the other environmental stressors, exposure to noise pollution from traffic and construction may plausibly aggravate hypertension in participants residing in overcrowded residential apartments of high building level density. A recent study reported that noise exposures exceeded acceptable levels in majority of the residential areas in Hong Kong [48], and the evidences of the associations between noise exposure and hypertension have been previously established [49,50].

Our finding with respect to liveable space is important in guiding housing policy in Hong Kong and other high-density cities with similar settings and population characteristics. Our results highlight the importance of optimizing housing provisioning and liveable space allocation, which continues to be one of the major social issues given the significant unmet demand for dwellings and high property prices. According to the 2014 statistics, the average price of private housing was US$127,816/100 square feet [51]. For public housing, the government’s flagship social housing scheme catering affordable housing to residents in the lower socioeconomic strata, the average monthly rent of public permanent housing was US$59.5/100 square feet [35]. Our subgroup analyses by housing type found that increments in floor area were associated with lower blood pressure outcomes, although the effect sizes were more pronounced among participants residing in public housing. This can further suggest a stress-related mechanism; adequate liveable space can plausibly ameliorate effects of social stress lowering hypertension among residents of lower socioeconomic position residing in overcrowded dense areas. Well-designed public housing with health-optimized liveable space and density can be the foundation of government’s housing policy for future-proofing population health. The observed negative association of liveable space among private housing residents may also be explained in terms of social stress amelioration. Furthermore, it is likely that private housing residents may be exposed to material deprivation, attributable to higher cost of rent and mortgage payments relative to income, consequently diminishing capacity to spend on healthy choices related to diet and lifestyle. The role of affordable housing achieved via housing policies that are well synchronized with macroeconomic determinants of market pricing in improving population health deserves further research.

That the associations of liveable space were more pronounced among female participants than in male may be related to time spent and utilization of these residential spaces [52]. The associations of liveable space were more pronounced among homemakers/students as compared to the other categories of employment status, given this subgroup is likely to spend more time at home, although there was no significant interaction effect.

At a neighborhood scale (macrolevel), we found that higher residential density was associated with 6.7% lower odds of hypertension, the result being significant only at baseline. It is plausible that higher residential density may be a proxy for walkable neighborhoods with greater access to community resources, thereby promoting physical activity through active living. Walkable communities are also associated with greater sense of community and social interactions, with a capacity to reduce social stress. Previous studies have reported beneficial associations of neighborhood-level residential density on obesity and physical activity [33,53,54]. The negative association of neighborhood walkability with hypertension has also been reported [10,11].

Our study found that in reference to participants who did not move, those moving to residences of lower liveable floor area was associated with higher odds of incident hypertension. It should be noted that the process of moving residences can be often disruptive. We also intended to examine this effect of downsizing in reference to participants who moved to residences of larger floor area. However, we did not have sufficient number of participants with hypertension cases (and statistical power) in the group moving residence of larger floor area to conduct such analysis. In Hong Kong, primary motivations for downsizing include desire to reside near employment centers or in the vicinity of public transit nodes (mass transit railway stations) where the rental costs and property prices are generally higher. Young families with 1 or more child entering primary school trade-off liveable space in exchange for residing in school catchment areas with relatively higher rental and property prices. It is also known that young adults sharing households with parents move out to live independently in smaller apartments. Our models could not control for the reasons for downsizing to residences of smaller floor area; nonetheless, we had adjusted for a range of sociodemographic variables.

Among the limitations, as in any observational study, it is difficult to establish causal inference, and residual confounding cannot be ruled out completely. Nonetheless, to examine robustness of our findings, we were able to develop models for change in blood pressure and incident hypertension, adjust for a range of confounders, and conduct sensitivity tests including propensity score analysis to examine the effect of moving from residence of higher to lower liveable space. The response rate of FAMILY Cohort across the 2 waves was 69.6%. Loss to follow-up was primarily attributable to death, emigration, loss of contact, or withdrawal from the study, which has the potential to introduce bias. Nonetheless, in our analytic samples, there were no systematic differences between participants at baseline and those who were successfully followed up in wave 2. Cohen’s w and d effect sizes consistently remained <0.2 (see Table 1). Furthermore, sensitivity test employing multiple imputation to account for loss to follow-up showed consistent results. The median follow-up duration of the present study was 2.17 years. Given that blood pressure outcomes are prone to fluctuations, future studies must be conducted to validate our findings over a longer follow-up period as well as employ more rigorous markers of cumulative vascular aging such as arterial stiffness that are less vulnerable to confounding by fluctuations [55]. Our models adjusted for number in the family, shared living, and housing type; however, our housing database could not account for subdivided housing, which could have potentially induced exposure misclassification. Subdivided housing is a unique characteristics of Hong Kong, given the significant unmet demand for housing and related market pressures. In old urban renewable areas, single-unit private apartments are further informally subdivided in to smaller units with no building level official records. According to the 2014 estimates from the Census and Statistics Department, there were 24,600 quarters with 86,400 subdivided units, accounting for 3.9% of all private/domestic quarters aged >25 years [56]. This may imply that if the participant resided in one of these units or building, our measured floor area may have been slightly overestimated and building unit density underestimated, so that our reported estimates of associations are likely to be conservative. Our models could not account for personal commute routes preferences, travel mode choices, and fares. The trade-offs between larger liveable space, albeit with poorer accessibility with longer and expensive commutes, require further consideration in future research. In Hong Kong’s context, such effects may be less likely to be significant, given the existence of one of the best public transport systems and government supported travel subsidies (such as the public transport fare subsidy, working family allowance, and the work incentive transport subsidy). The mean home-to-work trip time in Hong Kong has been comparatively low at 47 minutes [36]. In a Western context where densities are comparatively low, suburban densification is likely to be a public health opportunity if achieved by not only optimizing liveable household space and residential density, but also provisioning for adequate land use and employment mix, minimizing travel demand. We were able to adjust for shared living in our analyses. Future studies should further attempt to disentangle more nuanced effects of residing in households with multiple related families belonging to the same primary family versus cohabiting with members with no blood relationships such as friends or lodgers.

Our study has several strengths. We were able to leverage data from a Hong Kong–wide health cohort with data derived from 2 waves. The study was able to create highly characterized measures of housing environment, measured at participants’ residences or within a predefined neighborhood catchments of geocoded residence. To our knowledge, this is the first study to rigorously employ objective measures of housing environment at an individual level (liveable floor area, block level building density, and neighborhood-level residential density) to establish associations with hypertension, a key determinant of CVDs. Previous studies conducted thus far have used ecological scale design employing aggregate or self-reported data susceptible to measurement error and recall bias.

Hypertension is a silent killer, given that it is frequently asymptomatic and follow the rule of halves, whereby half of the population are undiagnosed, while of those treated, around half remain uncontrolled [4]. Household-level exposures are common risk factors falling in the etiologic pathway of a number of chronic diseases. Our findings suggest the importance of housing provisioning in terms of liveable space and residential density for population health. Given the large proportion of population with hypertension in Hong Kong, adequate housing in the form of provisioning of sufficient liveable space and creation of compact neighborhoods may be more relevant as population-wide preventive interventions. These interventions may shift the distribution of blood pressure in the population to the left, thereby lowering the risk of CVD and other related chronic diseases [57]. Our study linking housing-related metrics of crowding and density is the first, to our knowledge, to disentangle the differential associations with clinically measured health outcomes at micro–meso–macro scales. We could measure the association of liveable space upon hypertension controlling for densities at the building block and neighborhood levels that might otherwise confound an unadjusted model. With increasing urbanization and densification across the world, our findings that density effects function via different pathways producing opposing effects on hypertension in an extreme high-density setting (average residential density approaching 30,000 housing units per square kilometer in the current Hong Kong setting) can act as primary evidence for policy makers contemplating the appropriateness of high-density living solutions. Future studies are needed to verify the generalizability of our findings in cities of different population and density profiles and sociocultural contexts.

## Supporting information

S1 Appendix STROBE Checklist. STROBE, Strengthening the Reporting of Observational Studies in Epidemiology.
(DOCX)

S2 Appendix Supporting information tables. **Table A:** Specification of Western antihypertensive medication. **Table B:** Descriptive characteristics of participants of FAMILY Cohort at baseline and first follow-up for our cross-sectional analyses. **Table C:** Full models showing associations of housing environment exposures with prevalent and incident hypertension. **Table D:** Associations of liveable residential space (per 100 square feet increments) with blood pressure outcomes and hypertension among FAMILY Cohort participants aged 16 or above. **Table E:** Association of housing environment exposures with measures of blood pressure outcomes and hypertension in our target samples using multiple imputation to impute for missing observations across key covariates. **Table F:** Association of housing environment exposures with measures of blood pressure outcomes and hypertension for 37,656 participants using multiple imputation to impute for observations lost to follow-up and those with missingness across key covariates. **Table G:** Association of housing environment exposures with measures of blood pressure outcomes and prevalent hypertension among FAMILY Cohort participants aged 16 or above at baseline with built environment measured within 1-mile catchment (1,609 m). **Table H:** Association of housing environment exposures with measures of blood pressure outcomes and prevalent hypertension among FAMILY Cohort participants aged 16 or above in the first follow-up with built environment measured within 1-mile catchment (1,609 m). **Table I:** Association of housing environment exposures with blood pressure outcomes and incident hypertension among FAMILY Cohort participants aged 16 or above who were followed up and had not been diagnosed as hypertensives at baseline with built environment measured within 1-mile catchment (1,609 m). **Table J:** Associations of housing environment exposures with blood pressure outcomes and hypertension among FAMILY Cohort participants aged 16 or above, adjusting for household income. **Table K:** Associations of housing environment exposures with blood pressure outcomes and hypertension among FAMILY Cohort participants aged 16 or above, with age as a continuous variable. **Table L:** Association of liveable floor area and housing units per block with blood pressure outcomes by population subgroups of sex, age, income categories, employment status, and housing types for *n* = 20,244 participants at wave 2.
(DOCX)

S3 Appendix Supporting information figures. **Fig A:** An illustration showing the attributes of housing exposures in the developed HKHED database; livable floor area, building units per block and neighborhood residential density. km: kilometer. **Fig B:** Flowchart of the selection of participants for cross-sectional analyses at baseline and wave 2. **Fig C:** Flowchart of the selection of participants for longitudinal analyses on linked data across 2 waves. **Fig D:** Density plot showing the distribution of propensity scores of the incident hypertension model in the control group (participants who did not change their residential address between the 2 waves) marked as 0, and the treatment group (participants who changed residence to lower liveable floor area) marked as 1 after matching. HKHED, Hong Kong Housing Environment Database.
(DOCX)

## Abbreviations

β: beta
CI: confidence interval
CVD: cardiovascular disease
DALY: disability-adjusted life year
DBP: diastolic blood pressure
HA: Housing Authority
HKHED: Hong Kong Housing Environment Database
HS: Housing Society
IQR: interquartile range
MAP: mean arterial pressure
MAR: missing at random
MCAR: missing completely at random
MICE: multiple imputation by chained equations
MTR: mass transit railway
OR: odds ratio
SBP: systolic blood pressure
SD: standard deviation
STROBE: Strengthening the Reporting of Observational Studies in Epidemiology
WHO: World Health Organization
