# Supplementary material for: Liveable residential space, residential density, and hypertension in Hong Kong: A population-based cohort study
Source: PLoS Med. 2021 Nov 2;18(11):e1003824. doi: 10.1371/journal.pmed.1003824 (PMC8562807; doi:10.1371/journal.pmed.1003824)
Supplement: S1 Appendix — STROBE, Strengthening the Reporting of Observational Studies in Epidemiology. (DOCX) [file pmed.1003824.s001.docx]

**Liveable residential space, residential density and hypertension in Hong Kong: A population-based cohort study**

Chinmoy Sarkar^1,2^†*, Ka Yan Lai^1^†, Michael Y. Ni^1,2,3^, Sarika Kumari^1^, Gabriel M. Leung^2^, Chris Webster^1^

^1^*Healthy High Density Cities Lab, HKUrbanLab, The University of Hong Kong, Knowles Building, Pokfulam Road, Pokfulam, Hong Kong Special Administrative Region, China.*

*^2^School of Public Health, The University of Hong Kong, Patrick Manson Building, Sassoon Road, Pokfulam, Hong Kong Special Administrative Region, China.*

*^3^The State Key Laboratory of Brain and Cognitive Sciences, The University of Hong Kong, Hong Kong Special Administrative Region, China.*

*†These authors share first authorship on this work.*

**Corresponding author*

**Supporting information:**

S1 Appendix: STROBE Checklist

STROBE Statement presenting checklist of items that should be included in reports of cohort studies.

|  | Item No. | | Recommendation | Page  No. | Relevant text from manuscript |
| --- | --- | --- | --- | --- | --- |
| **Title and abstract** | 1 | | (*a*) Indicate the study’s design with a commonly used term in the title or the abstract |  | ‘Liveable residential space, residential density and hypertension in Hong Kong: A population-based cohort study’ |
|  |  |  | (*b*) Provide in the abstract an informative and balanced summary of what was done and what was found |  | Abstract |
| Introduction | | | | |  |
| Background/  rationale | 2 | | Explain the scientific background and rationale for the investigation being reported |  | Introduction |
| Objectives | 3 | | State specific objectives, including any pre-specified hypotheses |  | ‘The present study aims to investigate independent associations of objectively measured housing environment (liveable area and density) with blood pressure outcomes and risk of hypertension in the highly dense environmental setting of Hong Kong SAR. We also aim to explore effect modifications by age, sex, income, employment status and housing type and the potential impacts of moving to residences of smaller liveable area upon hypertension.’ |
| Methods | | | | |  |
| Study design | 4 | | Present key elements of study design early in the paper |  | Methods |
| Setting | 5 | | Describe the setting, locations, and relevant dates, including periods of recruitment, exposure, follow-up, and data collection |  | Methods, first paragraph |
| Participants | 6 | | (*a*) *Cohort study*—Give the eligibility criteria, and the sources and methods of selection of participants. Describe methods of follow-up  *Case-control study*—Give the eligibility criteria, and the sources and methods of case ascertainment and control selection. Give the rationale for the choice of cases and controls  *Cross-sectional study*—Give the eligibility criteria, and the sources and methods of selection of participants |  | Methods  Statistical analysis |
|  |  |  | (*b*) *Cohort study*—For matched studies, give matching criteria and number of exposed and unexposed  *Case-control study*—For matched studies, give matching criteria and the number of controls per case |  |  |
| Variables | 7 | | Clearly define all outcomes, exposures, predictors, potential confounders, and effect modifiers. Give diagnostic criteria, if applicable |  | Methods, Statistical analysis |
| Data sources/ measurement | 8* | | For each variable of interest, give sources of data and details of methods of assessment (measurement). Describe comparability of assessment methods if there is more than one group |  | Methods |
| Bias | 9 | | Describe any efforts to address potential sources of bias |  | Statistical analysis (second paragraph) |
| Study size | 10 | | Explain how the study size was arrived at |  | Results  Figs B and C in S3 Appendix |
| Quantitative variables | 11 | | Explain how quantitative variables were handled in the analyses. If applicable, describe which groupings were chosen and why |  | Statistical analysis |
| Statistical methods | 12 | | (*a*) Describe all statistical methods, including those used to control for confounding |  | Statistical analysis |
|  |  |  | (*b*) Describe any methods used to examine subgroups and interactions |  | Statistical analysis (second para) |
|  |  |  | (*c*) Explain how missing data were addressed |  | Statistical analysis (second para) |
|  |  |  | (*d*) *Cohort study*—If applicable, explain how loss to follow-up was addressed  *Case-control study*—If applicable, explain how matching of cases and controls was addressed  *Cross-sectional study*—If applicable, describe analytical methods taking account of sampling strategy |  | Statistical analysis (second para) |
|  |  |  | (*e*) Describe any sensitivity analyses |  | Statistical analysis (second para) |
| Results | | | | | |
| Participants | 13* | | (a) Report numbers of individuals at each stage of study—eg numbers potentially eligible, examined for eligibility, confirmed eligible, included in the study, completing follow-up, and analysed |  | Results |
|  |  |  | (b) Give reasons for non-participation at each stage |  | Figs B and C in S3 Appendix |
|  |  |  | (c) Consider use of a flow diagram |  | Figs B and C in S3 Appendix |
| Descriptive data | 14* | | (a) Give characteristics of study participants (eg demographic, clinical, social) and information on exposures and potential confounders |  | Results, Table 1 and Table B in S2 Appendix |
|  |  |  | (b) Indicate number of participants with missing data for each variable of interest |  | Results, Table 1 |
|  |  |  | (c) *Cohort study*—Summarise follow-up time (eg, average and total amount) |  | ‘…over the median follow-up period of 2.2 years (range: 1.3-3.6 years).’ |
| Outcome data | 15* | | *Cohort study*—Report numbers of outcome events or summary measures over time |  | Results (also see Tables 3, 4) |
|  |  |  | *Case-control study—*Report numbers in each exposure category, or summary measures of exposure |  |  |
|  |  |  | *Cross-sectional study—*Report numbers of outcome events or summary measures |  |  |
| Main results | 16 | | (*a*) Give unadjusted estimates and, if applicable, confounder-adjusted estimates and their precision (eg, 95% confidence interval). Make clear which confounders were adjusted for and why they were included |  | Results, Tables 2a-b, 3, 4 |
|  |  |  | (*b*) Report category boundaries when continuous variables were categorized |  |  |
|  |  |  | (*c*) If relevant, consider translating estimates of relative risk into absolute risk for a meaningful time period |  |  |
| Other analyses | 17 | | Report other analyses done—eg analyses of subgroups and interactions, and sensitivity analyses |  | Figures 1a-b, Table C in S2 Appendix  Tables 4, 5, and Tables E to L in S2 Appendix |
| Discussion | | | | | |
| Key results | | 18 | Summarise key results with reference to study objectives |  | Discussion, first paragraph |
| Limitations | | 19 | Discuss limitations of the study, taking into account sources of potential bias or imprecision. Discuss both direction and magnitude of any potential bias |  | Discussion |
| Interpretation | | 20 | Give a cautious overall interpretation of results considering objectives, limitations, multiplicity of analyses, results from similar studies, and other relevant evidence |  | Discussion |
| Generalisability | | 21 | Discuss the generalisability (external validity) of the study results |  | Discussion, last paragraph |
| Other information | | | | | |
| Funding | | 22 | Give the source of funding and the role of the funders for the present study and, if applicable, for the original study on which the present article is based |  | See funding section |

*Give information separately for cases and controls in case-control studies and, if applicable, for exposed and unexposed groups in cohort and cross-sectional studies.

**Note:** An Explanation and Elaboration article discusses each checklist item and gives methodological background and published examples of transparent reporting. The STROBE checklist is best used in conjunction with this article (freely available on the Web sites of PLoS Medicine at <http://www.plosmedicine.org/>, Annals of Internal Medicine at <http://www.annals.org/>, and Epidemiology at <http://www.epidem.com/>). Information on the STROBE Initiative is available at www.strobe-statement.org.
