# Supplementary material for: Liveable residential space, residential density, and hypertension in Hong Kong: A population-based cohort study
Source: PLoS Med. 2021 Nov 2;18(11):e1003824. doi: 10.1371/journal.pmed.1003824 (PMC8562807; doi:10.1371/journal.pmed.1003824)
Supplement: S2 Appendix — Table A: Specification of Western antihypertensive medication. Table B: Descriptive characteristics of participants of FAMILY Cohort at baseline and first follow-up for our cross-sectional analyses. Table C: Full models showing associations of housing environment exposures with prevalent and incident hypertension. Table D: Associations of liveable residential space (per 100 square feet increments) with blood pressure outcomes and hypertension among FAMILY Cohort participants aged 16 or above. Table E: Association of housing environment exposures with measures of blood pressure outcomes and hypertension in our target samples using multiple imputation to impute for missing observations across key covariates. Table F: Association of housing environment exposures with measures of blood pressure outcomes and hypertension for 37,656 participants using multiple imputation to impute for observations lost to follow-up and those with missingness across key covariates. Table G: Association of housing environment exposures with measures of blood pressure outcomes and prevalent hypertension among FAMILY Cohort participants aged 16 or above at baseline with built environment measured within 1-mile catchment (1,609 m). Table H: Association of housing environment exposures with measures of blood pressure outcomes and prevalent hypertension among FAMILY Cohort participants aged 16 or above in the first follow-up with built environment measured within 1-mile catchment (1,609 m). Table I: Association of housing environment exposures with blood pressure outcomes and incident hypertension among FAMILY Cohort participants aged 16 or above who were followed up and had not been diagnosed as hypertensives at baseline with built environment measured within 1-mile catchment (1,609 m). Table J: Associations of housing environment exposures with blood pressure outcomes and hypertension among FAMILY Cohort participants aged 16 or above, adjusting for household income. Table K: Associations of ho [file pmed.1003824.s002.docx]

**Liveable residential space, residential density and hypertension in Hong Kong: A population-based cohort study**

Chinmoy Sarkar^1,2^†*, Ka Yan Lai^1^†, Michael Y. Ni^1,2,3^, Sarika Kumari^1^, Gabriel M. Leung^2^, Chris Webster^1^

^1^*Healthy High Density Cities Lab, HKUrbanLab, The University of Hong Kong, Knowles Building, Pokfulam Road, Pokfulam, Hong Kong Special Administrative Region, China.*

*^2^School of Public Health, The University of Hong Kong, Patrick Manson Building, Sassoon Road, Pokfulam, Hong Kong Special Administrative Region, China.*

*^3^The State Key Laboratory of Brain and Cognitive Sciences, The University of Hong Kong, Hong Kong Special Administrative Region, China.*

*†These authors share first authorship on this work.*

**Corresponding author*

**Supporting information:**

S2 Appendix: Supporting Tables

**Table A: Specification of Western antihypertensive medication**

| **Generic name** | **Active substance/ ingredients** | **Brand names** |
| --- | --- | --- |
| Amedin | - | - |
| Amiloride | - | - |
| Amlodipine | Amlodipine besylate / amlodipine besilate (苯磺酸氨氯地平片) | Amlodipine-teva |
|  |  | Norvasc (健壓樂) |
|  |  | Amlong-5 |
|  | Amlodipine besylate and Atorvastatin calcium | Caduet (確健心) |
|  | Amlodipine and atenolol | Amlong-A |
| Atenolol | Atenolol | Apo-atenol |
|  |  | Totamol |
|  |  | Atenolol Actavis |
| Bisoprolol and derivates | Bisoprolol hemifumarate and hydrochlorothiazide | Lodoz |
|  | Bisoprolol fumarate | Concor |
| Candesartan | Candesartan cilexetil | Blopress (博脈舒) |
| Captopril | - | - |
| Carvedilol | - | - |
| Diltiazem | Diltiazem HCl | Herbesser |
|  |  | Cartia XT |
| Doxazosin | Doxazosin mesylate | Cardura |
|  |  | Cardura XL |
|  |  | Carduran |
| Enalapril | Enalapril maleate | Lapril |
|  |  | Renitec (悅寧定) |
| Felodipine | Felodipine | Plendil |
| Furosemide | Furosemide | Frusemide |
| Glyceryl trinitrate | - | - |
| Hydralazine | Hydralazine hydrochloride | - |
| Hydrochlorothiazide | Hydrochlorothiazide and triamterene | Dyazide |
| Indapamide | Indapamide hemihydrate (Indapamide SR) | Natrilix SR |
| Irbesartan and derivatives | Irbesartan | Aprovel (安博維) |
|  | Irbesartan and hydrochlorothiazide | CoAprovel |
| Labetalol | - | - |
| Lacidipine | Lacidipine | Lacibloc |
|  |  | Lacipil |
| Lisinopril | Lisinopril dihydrate | Zestril |
| Lorsartan | lorsartan potassium | Cozaar (科素亞) |
|  | Losartan potassium and hydrochlorothiazide | Hyzaar (海捷亞) |
| Methyldopa | - | - |
| Metoprolol and derivatives, e.g. metoprolol tartrate | Metoprolol succinate | Betaloc ZOK |
|  |  | Metoprolol CR/XL |
|  | Metoprolol tartrate (酒石酸美托洛尔) | Betaloc |
| Napamide | - | - |
| Nifedipine | Nifedipine | Nifedi-Denk |
|  |  | Adalat |
|  |  | Adalat retard (拜新同) |
|  |  | Nifedipine SR |
| Olmesartan | Olmesartan medoxomil and hydrochlorothiazide | Olmetec Plus (安脈加) |
| Perindopril | Perindopril arginine | Acertil |
|  | Perindopril tert-butylamine | Perindal |
| Prazosin | Prazosin hydrochloride (HCl) | - |
| Propranolol | Propranolol hydrochloride | Inderal |
| Ramipril | Ramipril | Tritace |
| Spironolactone | - | - |
| Telmisartan | Telmisartan | Micardis |
| Terazosin | Terazosin hydrochloride | - |
| Valsartan | Valsartan | Diovan |
|  | Valsartan and hydrochlorothiazide | Co-Diovan |
| Anti-hypertensive, unspecified | 血壓  血壓丸  血壓藥  降血壓  Blood pressure | |

**Table B: Descriptive characteristics of participants of FAMILY Cohort at baseline and first follow-up for our cross-sectional analyses.**

| **Participant characteristics** | **Baseline (N=37,656)** | **Wave 2 (N=25,209)** |
| --- | --- | --- |
| *Covariates:* |  |  |
| Age in years (Mean, SD) | 47.0 (17.6) | 48.6 (17.9) |
| Gender N (%): Female | 20,537 (54.5) | 13,848 (54.9) |
| Male | 17,119 (45.5) | 11,361 (45.1) |
| Highest educational qualification N (%): Primary | 9,709 (25.8) | 6,384 (25.3) |
| Secondary | 17,971 (47.7) | 11,481 (45.5) |
| Tertiary | 9,794 (26.0) | 7,307 (29.0) |
| Missing | 182 (0.5) | 37 (0.2) |
| Marital status N (%): Never married | 9,270 (24.6) | 5,879 (23.3) |
| Married | 24,830 (65.9) | 16,578 (65.8) |
| Widowed/Divorced/separated | 3,502 (9.3) | 2,718 (10.8) |
| Missing | 54 (0.1) | 34 (0.1) |
| Employment status N (%): Employed | 18,186 (48.3) | 11,958 (47.4) |
| Home maker, students and others | 10,681 (28.4) | 7,043 (27.9) |
| Retiree/unemployed | 5,140 (13.7) | 3,609 (14.3) |
| Missing | 3,649 (9.7) | 2,599 (10.3) |
| Personal income (HK$): <4,999 | 16,046 (42.6) | 10,427 (41.4) |
| 5,000-9,999 | 6,749 (17.9) | 4,279 (17.0) |
| 10,000-14,999 | 5,316 (14.1) | 3,886 (15.4) |
| ≥15,000 | 6,735 (17.9) | 5,503 (21.8) |
| Missing | 2,810 (7.5) | 1,114 (4.4) |
| Smoking status N (%): Nonsmoker/past smoker | 32,198 (85.5) | 22,236 (88.2) |
| Current smoker | 5,418 (14.4) | 2,938 (11.7) |
| Missing | 40 (0.1) | 35 (0.1) |
| Alcohol consumption N (%): Never/former drinker | 28,481 (75.6) | 19,564 (77.6) |
| Occasional/1-3 per month | 6,200 (16.5) | 4,120 (16.3) |
| 1-3 per week to daily | 2,734 (7.3) | 1,477 (5.9) |
| Missing | 241 (0.6) | 48 (0.2) |
| Number of family members N (%): living alone | 5,422 (14.4) | 3,191 (12.7) |
| 2 | 12,072 (32.1) | 8,902 (35.3) |
| 3 | 9,311 (24.7) | 6,216 (24.7) |
| 4 | 7,723 (20.5) | 4,857 (19.3) |
| ≥5 | 3,128 (8.3) | 2,043 (8.1) |
| Number of core family N (%): Single family | 22,579 (60.0) | 15,575 (61.8) |
| Two families | 6,099 (16.2) | 4,344 (17.2) |
| Three families | 3,860 (10.3) | 2,553 (10.1) |
| >Three families | 5,118 (13.6) | 2,737 (10.9) |
| Overweight or obese N (%):BMI<23 | 17,528 (46.6) | 11,260 (44.7) |
| BMI≥23 | 19,422 (51.6) | 13,161 (52.2) |
| Missing | 706 (1.9) | 788 (3.1) |
| Self-reported coronary heart disease N (%): No | 36,346 (96.5) | 24,415 (96.9) |
| Yes | 1,054 (2.8) | 650 (2.6) |
| Missing | 256 (0.7) | 144 (0.6) |
| *Outcomes* |  |  |
| Diastolic blood pressure, mmHg (Mean, SD) | 78.7 (11.4) | 76.2 (10.9) |
| Systolic blood pressure, mmHg (Mean, SD) | 125.9 (20.5) | 121.6 (19.0) |
| Mean Arterial pressure, mmHg (Mean, SD) | 94.4 (13.5) | 91.3 (12.6) |
| *Built environment variables* |  |  |
| Floor area, square feet (Mean, SD) | 458.4 (160.1) | 454.1 (156.0) |
| Housing units per block (Mean, SD) | 470.4 (302.2) | 473.4 (301.4) |
| Residential unit density within 0.5 mile (units/Km^2^) (Mean, SD) | 28,850.0 (11,225.9) | 29,427.9 (11,103.0) |
| Residential unit density within 1 mile (units/Km^2^) (Mean, SD) | 21,381.5 (10,128.3) | 21,629.2 (8,731.1) |
| Housing type N (%): Public housing | 17,856 (47.4) | 11,675 (46.3) |
| Private housing/subsidized sale flats | 19,799 (52.6) | 12,781 (50.7) |
| Missing | 1 (0.0) | 753 (3.0) |
| Floor level (Mean, SD) | 15.8 (10.4) | 16.1 (10.7) |
| Public transport density within 0.5 mile (units/Km^2^) (Mean, SD) | 41.2 (21.3) | 40.8 (21.1) |
| Public transport density within 1 mile (units/Km^2^) (Mean, SD) | 35.7 (17.7) | 35.4 (17.5) |
| Terrain variability within 0.5 mile (SD, degrees) (Mean, SD) | 10.7 (4.8) | 10.7 (4.9) |
| Terrain variability within 1 mile (SD, degrees) (Mean, SD) | 11.8 (4.2) | 11.8 (4.2) |

BMI: body mass index, Km: kilometre, SD: standard deviation.

**Table C: Full models showing associations of housing environment exposures with prevalent and incident hypertension**

|  | Prevalent hypertension (baseline) | Prevalent hypertension (first follow-up) | Incident hypertension (first follow-up) |
| --- | --- | --- | --- |
|  | N= 30,439 | N=20,244 | N=13,895 |
|  | OR (95% CI) p-value | OR (95% CI) p-value | OR (95% CI) p-value |
| *Housing environment* |  |  |  |
| Floor area, square feet (per IQR) | 0.955 (0.918,0.993) 0.022 | 0.932 (0.884,0.983) 0.009 | 0.909 (0.836,0.988) 0.025 |
| Housing units per block (per IQR) | 1.091 (1.024,1.162) 0.007 | 1.068 (0.984,1.159) 0.118 | 1.030 (0.905,1.172) 0.657 |
| Neighbourhood residential density, units/Km^2^ (per IQR) | 0.933 (0.899,0.969) <0.001 | 1.016 (0.968,1.068) 0.518 | 0.981 (0.906,1.062) 0.634 |
| *Other neighbourhood environment* |  |  |  |
| Housing floor level | 1.000 (0.997,1.003) 0.803 | 0.998 (0.994,1.002) 0.269 | 1.001 (0.995,1.007) 0.777 |
| Terrain variability (Slope SD) | 0.999 (0.993,1.005) 0.780 | 0.998 (0.99,1.006) 0.582 | 0.991 (0.978,1.003) 0.141 |
| Density of public transit (0.5 mile) | 1.034 (0.998,1.071) 0.064 | 1.022 (0.977,1.069) 0.348 | 1.030 (0.958,1.107) 0.422 |
| *Socio-demographic covariates* |  |  |  |
| Age in years (Ref: ≤40) |  |  |  |
| 40-50 | 2.456 (2.217,2.722) <0.001 | 2.082 (1.781,2.435) <0.001 | 1.831 (1.482,2.264) <0.001 |
| 50-60 | 3.974 (3.572,4.422) <0.001 | 3.200 (2.733,3.746) <0.001 | 3.247 (2.614,4.032) <0.001 |
| >60 | 7.336 (6.503,8.274) <0.001 | 6.303 (5.318,7.469) <0.001 | 5.827 (4.543,7.476) <0.001 |
| Sex (Ref: Male) |  |  |  |
| Female | 0.760 (0.709,0.815) <0.001 | 0.782 (0.712,0.86) <0.001 | 0.835 (0.718,0.971) 0.019 |
| Marital status (Ref: Never married) |  |  |  |
| Married | 0.973 (0.878,1.078) 0.600 | 0.946 (0.814,1.099) 0.469 | 0.934 (0.746,1.170) 0.553 |
| Widowed/Divorced /separated | 1.109 (0.968,1.271) 0.135 | 1.114 (0.926,1.34) 0.251 | 1.183 (0.889,1.575) 0.249 |
| Personal income in HK$ (Ref: ≤4,999) | |  |  |
| 5,000-9,999 | 0.900 (0.819,0.989) 0.028 | 0.913 (0.809,1.031) 0.142 | 0.893 (0.736,1.083) 0.248 |
| 10,000-14,999 | 0.937 (0.839,1.046) 0.248 | 0.968 (0.838,1.119) 0.664 | 0.954 (0.765,1.190) 0.678 |
| ≥15,000 | 0.921 (0.819,1.035) 0.168 | 1.011 (0.871,1.173) 0.886 | 1.142 (0.912,1.429) 0.248 |
| Highest educational qualification (Ref: Primary) | | | |
| Secondary | 0.858 (0.797,0.923) <0.001 | 0.746 (0.679,0.821) <0.001 | 0.742 (0.639,0.863) <0.001 |
| Tertiary or above | 0.743 (0.668,0.827) <0.001 | 0.636 (0.554,0.732) <0.001 | 0.655 (0.526,0.815) <0.001 |
| Employment status (Ref: Employed) | | | |
| Homemaker/student/others | 1.086 (0.997,1.182) 0.059 | 0.973 (0.866,1.094) 0.648 | 0.953 (0.798,1.139) 0.596 |
| Retiree/unemployed | 1.173 (1.047,1.313) 0.006 | 1.118 (0.971,1.287) 0.122 | 1.195 (0.958,1.490) 0.114 |
| *Lifestyle and comorbidities* |  |  |  |
| Number in family (Ref: Living alone) | | | |
| 2 | 0.682 (0.622,0.748) <0.001 | 0.881 (0.776,1.000) 0.050 | 0.918 (0.748,1.126) 0.411 |
| 3 | 0.621 (0.564,0.685) <0.001 | 0.785 (0.685,0.900) 0.001 | 0.813 (0.653,1.013) 0.066 |
| 4 | 0.617 (0.556,0.684) <0.001 | 0.758 (0.654,0.879) <0.001 | 0.812 (0.641,1.029) 0.085 |
| ≥5 | 0.558 (0.488,0.639) <0.001 | 0.787 (0.653,0.948) 0.012 | 0.832 (0.621,1.115) 0.218 |
| Shared living (Ref: Single-family) |  |  |  |
| Two family | 0.927 (0.853,1.007) 0.071 | 0.911 (0.82,1.012) 0.084 | 0.926 (0.786,1.090) 0.356 |
| Three family | 1.000 (0.905,1.105) 0.995 | 0.829 (0.723,0.950) 0.007 | 0.874 (0.707,1.082) 0.216 |
| >Three family | 0.957 (0.875,1.045) 0.327 | 0.966 (0.849,1.099) 0.601 | 0.876 (0.712,1.078) 0.211 |
| Housing type (Ref: Public housing) |  |  |  |
| Private housing/subsidized sale flats | 0.954 (0.881,1.033) 0.244 | 1.033 (0.931,1.147) 0.539 | 1.080 (0.916,1.272) 0.359 |
| Smoking status (Ref: Non-smoker/past smoker) | | | |
| Smoker | 1.019 (0.932,1.114) 0.680 | 1.129 (0.995,1.28) 0.060 | 1.055 (0.868,1.282) 0.591 |
| Alcohol intake frequency (Ref: Never/former drinker) | | | |
| Occasional up to 1-3 per month | 0.917 (0.843,0.997) 0.043 | 0.996 (0.893,1.11) 0.938 | 1.020 (0.861,1.208) 0.823 |
| 1-3 per week up to daily | 1.058 (0.945,1.184) 0.326 | 1.126 (0.963,1.317) 0.137 | 1.185 (0.939,1.496) 0.154 |
| Body mass index (Ref: Normal weight) | | | |
| Overweight/weight | 2.518 (2.368,2.678) <0.001 | 2.584 (2.376,2.811) <0.001 | 2.498 (2.188,2.852) <0.001 |
| Coronary heart disease (Yes vs. No) | 0.900 (0.761,1.065) 0.220 | 1.278 (1.022,1.598) 0.031 | 1.619 (1.146,2.286) 0.006 |
| Log follow-up time | - | - | 5.497 (3.771,8.014) <0.001 |

IQR: interquartile range, Km: Kilometre, OR: odds ratio, CI: confidence interval

**Table D. Associations of liveable residential space (per 100 square feet increments) with blood pressure outcomes and hypertension among FAMILY Cohort participants aged 16 or above**

| **Floor area, square feet**  (per 100 square feet increment)^a^ | **Diastolic blood pressure (mm Hg)** | **Systolic blood pressure (mm Hg)** | **Mean arterial pressure (mm Hg)** | **Hypertension** |
| --- | --- | --- | --- | --- |
|  | β (95% CI) p-value | β (95% CI) p-value | β (95% CI) p-value | OR (95% CI) p-value |
| **Cross sectional model at baseline,** n=30,439 | -0.147 (-0.229,-0.065) <0.001 | -0.173 (-0.301,-0.046) 0.008 | -0.156 (-0.246,-0.065) <0.001 | -0.975 (0.954,0.996) 0.022 |
| **Cross sectional model at wave 2**, n=20,244 | -0.175 (-0.271,-0.079) <0.001 | -0.237 (-0.386,-0.088) 0.002 | -0.195 (-0.299,-0.092) <0.001 | 0.962 (0.935,0.991) 0.009 |
| **Longitudinal model of incident outcomes**, n=13,895 | -0.130 (-0.236,-0.024) 0.016 | -0.140 (-0.289,0.009) 0.065 | -0.133 (-0.243,-0.023) 0.017 | 0.949 (0.907,0.993) 0.025 |

^a^Models represent fully-adjusted accounting for socio-demographics (age, sex, marital status, employment status, educational attainment, household income), lifestyle (smoking status, alcohol intake frequency, number of family members, shared living, housing type), comorbidities (obesity, cardiac heart disease), residential density (housing units per block and neighbourhood residential density within a 0.5-mile street catchment) and environment (housing floor level, density of public transport and terrain, the latter two within a 0.5-mile buffer). The longitudinal models included participants who were followed-up and were non-hypertensive at baseline and additionally adjusted for logarithm of follow-up time.

OR: odds ratio, β: beta, CI: confidence interval.

**Table E. Association of housing environment exposures with measures of blood pressure outcomes and hypertension in our target samples using multiple imputation to impute for missing observations across key covariates.**

| **Housing environment^a^** | **Diastolic blood pressure**  **(mm Hg)** | **Systolic blood pressure**  **(mm Hg)** | **Mean arterial pressure**  **(mm Hg)** | **Hypertension** |
| --- | --- | --- | --- | --- |
|  | β (95% CI) p-value | β (95% CI) p-value | β (95% CI) p-value | OR (95% CI) p-value |
| **Cross sectional model at baseline, N=37,656**^a^ |  |  |  |  |
| Floor area, square feet (per IQR) | -0.271 (-0.405, -0.137) <0.001 | -0.364 (-0.576, -0.152) 0.001 | -0.302 (-0.451, -0.153) <0.001 | 0.958 (0.925, 0.992) 0.017 |
| Housing units per block (per IQR) | 0.323 (0.084, 0.562) 0.008 | 0.464 (0.077, 0.850) 0.019 | 0.370 (0.101, 0.639) 0.007 | 1.054 (0.997, 1.115) 0.063 |
| Neighbourhood residential density, units/Km^2^ (0.5 mi, per IQR) | -0.226 (-0.363, -0.089) 0.001 | -0.264 (-0.485, -0.043) 0.019 | -0.239 (-0.393, -0.085) 0.002 | 0.962 (0.931, 0.995) 0.023 |
|  |  |  |  |  |
| **Cross sectional model at Wave 2, N=25,209**^a^ |  |  |  |  |
| Floor area, square feet (per IQR) | -0.341 (-0.503, -0.179) <0.001 | -0.458 (-0.711, -0.206) <0.001 | -0.380 (-0.555, -0.205) <0.001 | 0.923 (0.880, 0.968) 0.001 |
| Housing units per block (per IQR) | 0.396 (0.123, 0.669) 0.004 | 0.494 (0.054, 0.933) 0.028 | 0.428 (0.125, 0.732) 0.006 | 1.031 (0.961, 1.107) 0.395 |
| Neighbourhood residential density, units/Km^2^ (0.5 mi, per IQR) | -0.031 (-0.192, 0.131) 0.710 | -0.180 (0.438, 0.078) 0.171 | -0.080 (-0.260, 0.099) 0.381 | 1.020 (0.977, 1.065) 0.361 |
|  |  |  |  |  |
| **Longitudinal model of incident outcomes, N=16,388**^a^ |  |  |  |  |
| Floor area, square feet (per IQR) | -0.250 (-0.431, -0.068) 0.007 | -0.235 (-0.493, 0.023) 0.074 | -0.245 (-0.434, -0.055) 0.011 | 0.910 (0.844, 0.981) 0.014 |
| Housing units per block (per IQR) | 0.320 (0.018, 0.622) 0.038 | 0.476 (0.023, 0.929) 0.039 | 0.372 (0.048, 0.696) 0.024 | 1.007 (0.899, 1.129) 0.899 |
| Neighbourhood residential density, units/Km^2^ (0.5 mi, per IQR) | 0.058 (-0.119, 0.234) 0.523 | -0.128 (-0.397, 0.140) 0.348 | -0.004 (-0.196, 0.187) 0.964 | 0.990 (0.923, 1.063) 0.791 |

^a^The imputation model only imputed missingness across covariates in the cross sectional and longitudinal models and all covariates, and exposure and outcome variables were included. A total of 20 imputation sets were created. Fully-adjusted models accounting for socio-demographics (age, sex, marital status, employment status, educational attainment, income), lifestyle (smoking status, alcohol intake frequency, number of family members, shared living, housing type), comorbidities (obesity, cardiac heart disease) and environment (housing floor level, density of public transport and terrain within 0.5-mile catchment) and logarithm of follow-up time (for longitudinal models only). IQR: interquartile range, Km: Kilometre, mi: mile, OR: odds ratio, CI: confidence interval.

**Table F. Association of housing environment exposures with measures of blood pressure outcomes and hypertension for 37,656 participants using multiple imputation to impute for observations lost to follow-up and those with missingness across key covariates.**

| **Housing environment^a^** | **Diastolic blood pressure**  **(mm Hg)** | **Systolic blood pressure**  **(mm Hg)** | **Mean arterial pressure**  **(mm Hg)** | **Hypertension** |
| --- | --- | --- | --- | --- |
|  | β (95% CI) p-value | β (95% CI) p-value | β (95% CI) p-value | OR (95% CI) p-value |
| **Cross sectional model at baseline**^a^ |  |  |  |  |
| Floor area, square feet (per IQR) | -0.270 (-0.405,-0.136) <0.001 | -0.363 (-0.574,-0.151) 0.001 | -0.301 (-0.449,-0.153) <0.001 | 0.958 (0.925,0.992) 0.016 |
| Housing units per block (per IQR) | 0.324 (0.085,0.563) 0.008 | 0.464 (0.077,0.850) 0.019 | 0.371 (0.102,0.640) 0.007 | 1.054 (0.997,1.114) 0.064 |
| Neighbourhood residential density, units/Km^2^ (0.5 mi, per IQR) | -0.226 (-0.362,-0.089) 0.001 | -0.263 (-0.484,-0.042) 0.020 | -0.238 (-0.392,-0.084) 0.002 | 0.963 (0.931,0.995) 0.024 |
|  |  |  |  |  |
| **Cross sectional model at Wave 2**^a^ |  |  |  |  |
| Floor area, square feet (per IQR) | -0.329 (-0.522,-0.137) 0.001 | -0.500 (-0.761,-0.238) <0.001 | -0.386 (-0.586,-0.187) <0.001 | 0.920 (0.875,0.966) 0.001 |
| Housing units per block (per IQR) | 0.467 (0.162,0.773) 0.003 | 0.480 (0.002,0.959) 0.049 | 0.472 (0.131,0.812) 0.007 | 1.021 (0.948,1.100) 0.579 |
| Neighbourhood residential density, units/Km^2^ (0.5 mi, per IQR) | -0.006 (-0.178,0.166) 0.946 | -0.186 (-0.454,0.082) 0.173 | -0.066 (-0.256,0.124) 0.494 | 1.019 (0.976,1.064) 0.392 |
|  |  |  |  |  |
| **Longitudinal model of incident outcomes**^a^ |  |  |  |  |
| Floor area, square feet (per IQR) | -0.252 (-0.453,-0.052) 0.014 | -0.317 (-0.584,-0.049) 0.021 | -0.274 (-0.479,-0.068) 0.010 | 0.907 (0.847,0.971) 0.005 |
| Housing units per block (per IQR) | 0.340 (0.036,0.645) 0.029 | 0.414 (-0.075,0.903) 0.097 | 0.365 (0.024,0.706) 0.036 | 0.996 (0.884,1.123) 0.953 |
| Neighbourhood residential density, units/Km^2^ (0.5 mi, per IQR) | 0.034 (-0.153,0.221) 0.721 | -0.146 (-0.417,0.126) 0.290 | -0.026 (-0.228,0.175) 0.797 | 1.012 (0.948,1.081) 0.710 |

^a^The imputation model imputed both missingness across covariates and loss to follow-up in the cross sectional and longitudinal models and comprised all covariates, and exposure and outcome variables at baseline and wave 2 as input. A total of 20 imputation sets were created. Fully-adjusted models accounting for socio-demographics (age, sex, marital status, employment status, educational attainment, income), lifestyle (smoking status, alcohol intake frequency, number of family members, shared living, housing type), comorbidities (obesity, cardiac heart disease) and environment (housing floor level, density of public transport and terrain within 0.5-mile catchment) and logarithm of follow-up time (for longitudinal models only). IQR: interquartile range, Km: Kilometre, mi: mile, OR: odds ratio, β: beta, CI: confidence interval.

**Table G. Association of housing environment exposures with measures of blood pressure outcomes and prevalent hypertension among FAMILY Cohort participants aged 16 or above at baseline with built environment measured within 1-mile catchment (1,609 m).**

| **Housing environment^a^** | **Diastolic blood**  **pressure**  **(mm Hg)** | **Systolic blood pressure**  **(mm Hg)** | **Mean arterial pressure**  **(mm Hg)** | **Hypertension** |
| --- | --- | --- | --- | --- |
|  | β (95% CI) p-value | β (95% CI) p-value | β (95% CI) p-value | OR (95% CI) p-value |
| **Model 1^b^,** n=37,656 |  |  |  |  |
| Floor area, square feet (per IQR) | -0.602 (-0.734,-0.469) <0.001 | -1.122 (-1.330,-0.914) <0.001 | -0.775 (-0.923,-0.627) <0.001 | 0.882 (0.853,0.912) <0.001 |
| Housing units per block (per IQR) | 0.580 (0.387,0.772) <0.001 | 0.804 (0.494,1.114) <0.001 | 0.655 (0.438,0.871) <0.001 | 1.082 (1.037,1.129) <0.001 |
| Neighbourhood residential density, units/Km^2^ (1 mi, per IQR) | -0.222 (-0.338,-0.106) <0.001 | -0.196 (-0.393,0.001) 0.051 | -0.214 (-0.347,-0.080) 0.002 | 0.971 (0.944,0.998) 0.034 |
|  |  |  |  |  |
| **Model 2^c^,** N=30,439 |  |  |  |  |
| Floor area, square feet (per IQR) | -0.279 (-0.429,-0.130) <0.001 | -0.325 (-0.557,-0.092) 0.006 | -0.294 (-0.459,-0.129) 0.001 | 0.954 (0.917,0.992) 0.018 |
| Housing units per block (per IQR) | 0.405 (0.143,0.667) 0.002 | 0.649 (0.226,1.072) 0.003 | 0.486 (0.191,0.782) 0.001 | 1.076 (1.011,1.145) 0.021 |
| Neighbourhood residential density, units/Km^2^ (1 mi, per IQR) | -0.240 (-0.372,-0.107) <0.001 | -0.234 (-0.464,-0.004) 0.046 | -0.238 (-0.392,-0.083) 0.003 | 0.957 (0.924,0.990) 0.012 |
|  |  |  |  |  |

^a^Model with neighbourhood environment (residential density, density of public transport and terrain) measured within 1-mile (1,609m) street catchment of geocoded participants’ residence.

^b^ Models adjusting for age and sex.

^c^Fully-adjusted models accounting for socio-demographics (age, sex, marital status, employment status, educational attainment, income), lifestyle (smoking status, alcohol intake frequency, number of family members, shared living, housing type), comorbidities (obesity, cardiac heart disease) and environment (housing floor level, density of public transport and terrain).

IQR: interquartile range, Km: Kilometre, mi: mile, OR: odds ratio, β: beta, CI: confidence interval.

**Table H**. **Association of housing environment exposures with measures of blood pressure outcomes and prevalent hypertension among FAMILY Cohort participants aged 16 or above in the first follow-up with built environment measured within 1-mile catchment (1,609 m).**

| **Housing environment^a^** | **Diastolic blood pressure**  **(mm Hg)** | **Systolic blood**  **pressure**  **(mm Hg)** | **Mean arterial**  **pressure**  **(mm Hg)** | **Hypertension** |
| --- | --- | --- | --- | --- |
|  | β (95% CI) p-value | β (95% CI) p-value | β (95% CI) p-value | OR (95% CI) p-value |
| **Model 1^b^,** n=25,209 |  |  |  |  |
| Floor area, square feet (per IQR) | -0.533 (-0.691,-0.374) <0.001 | -1.013 (-1.263,-0.763) <0.001 | -0.693 (-0.867,-0.519) <0.001 | 0.864 (0.827,0.903) <0.001 |
| Housing units per block (per IQR) | 0.606 (0.383,0.829) <0.001 | 0.750 (0.395,1.106) <0.001 | 0.654 (0.406,0.902) <0.001 | 1.054 (0.998,1.112) 0.057 |
| Neighbourhood residential density, units/Km^2^ (1 mi, per IQR) | -0.091 (-0.252,0.069) 0.265 | -0.136 (-0.391,0.118) 0.294 | -0.106 (-0.286,0.073) 0.246 | 1.015 (0.975,1.057) 0.459 |
|  |  |  |  |  |
| **Model 2^c^,** N=20,244 |  |  |  |  |
| Floor area, square feet (per IQR) | -0.323 (-0.498,-0.148) <0.001 | -0.442 (-0.713,-0.17) 0.001 | -0.362 (-0.551,-0.174) <0.001 | 0.931 (0.883,0.981) 0.007 |
| Housing units per block (per IQR) | 0.456 (0.154,0.758) 0.003 | 0.639 (0.155,1.123) 0.010 | 0.517 (0.180,0.854) 0.003 | 1.077 (0.993,1.168) 0.073 |
| Neighbourhood residential density, units/Km^2^ (1 mi, per IQR) | -0.015 (-0.204,0.174) 0.874 | -0.106 (-0.415,0.204) 0.504 | -0.045 (-0.26,0.169) 0.678 | 0.991 (0.941,1.045) 0.743 |
|  |  |  |  |  |

^a^Model with neighbourhood environment (residential density, density of public transport and terrain) measured within 1-mile (1,609m) street catchment of geocoded participants’ residence.

^b^Models adjusting for age and sex.

^c^Fully-adjusted models accounting for socio-demographics (age, sex, marital status, employment status, educational attainment, income), lifestyle (smoking status, alcohol intake frequency, number of family members, shared living, housing type), comorbidities (obesity, cardiac heart disease) and other environment (housing floor level, density of public transport and terrain).

IQR: interquartile range, Km: Kilometre mi: mile, OR: odds ratio, β: beta, CI: confidence interval.

**Table I. Association of housing environment exposures with blood pressure outcomes and incident hypertension among FAMILY Cohort participants aged 16 or above who were followed-up and had not been diagnosed as hypertensives at baseline with built environment measured within 1-mile catchment (1,609 m).**

| **Housing environment^a^** | **Diastolic blood pressure**  **(mm Hg)**  β (95% CI) p-value | **Systolic blood pressure**  **(mm Hg)**  β (95% CI) p-value | **Mean arterial pressure**  **(mm Hg)**  β (95% CI) p-value | **Incident hypertension***  β (95% CI) p-value |
| --- | --- | --- | --- | --- |
| **Model 1**^b^, n=16,390 |  |  |  |  |
| Floor area, square feet (per IQR) | -0.366 (-0.542,-0.190) <0.001 | -0.545 (-0.799,-0.290) <0.001 | -0.426 (-0.613,-0.239) <0.001 | 0.875 (0.815,0.938) <0.001 |
| Housing units per block (per IQR) | 0.404 (0.157,0.652)  0.001 | 0.679 (0.313,1.045) <0.001 | 0.496 (0.230,0.762) <0.001 | 1.017 (0.931,1.111) 0.711 |
| Neighbourhood residential density, units/Km^2^ (1 mi, per IQR) | -0.008 (-0.180,0.163) 0.923 | -0.139 (-0.399,0.122) 0.297 | -0.052 (-0.238,0.135) 0.586 | 0.975 (0.911,1.043) 0.461 |
|  |  |  |  |  |
| **Model 2**^c^, n=13,897 |  |  |  |  |
| Floor area, square feet (per IQR) | -0.234 (-0.427,-0.041) 0.017 | -0.252 (-0.524,0.019) 0.069 | -0.240 (-0.440,-0.040) 0.019 | 0.908 (0.837,0.986) 0.022 |
| Housing units per block (per IQR) | 0.385 (0.061,0.709)  0.020 | 0.637 (0.148,1.126) 0.011 | 0.469 (0.120,0.818) 0.008 | 1.045 (0.920,1.186) 0.501 |
| Neighbourhood residential density, units/Km^2^ (1 mi, per IQR) | -0.234 (-0.427,-0.041) 0.017 | -0.137 (-0.459,0.184) 0.402 | -0.035 (-0.259,0.190) 0.762 | 0.931 (0.851,1.020) 0.125 |

^a^Model with neighbourhood environment (residential density, density of public transport and terrain) measured within 1-mile (1,609m) street catchment of geocoded participants’ residence.

^b^Models adjusting for age at baseline. sex and logarithm of follow-up time.

^c^Fully-adjusted models accounting for socio-demographics (age, sex, marital status, employment status, educational attainment, income), lifestyle (smoking status, alcohol intake frequency, number of family members, shared living, housing type), comorbidities (obesity, cardiac heart disease) and environment (housing floor level, density of public transport and terrain) and logarithm of follow-up time.

*****The number of hypertension cases in the fully-adjusted model was 1,333 and cumulative incidence was 9.6%.

IQR: interquartile range, Km: Kilometre mi: mile, β: beta, CI: confidence interval.

**Table J. Associations of housing environment exposures with blood pressure outcomes and hypertension among FAMILY Cohort participants aged 16 or above, adjusting for household income**

| **Housing environment^a^** | **Diastolic blood pressure (mm Hg)** | **Systolic blood pressure (mm Hg)** | **Mean arterial pressure (mm Hg)** | **Incident hypertension*** |
| --- | --- | --- | --- | --- |
|  | β (95% CI) p-value | β (95% CI) p-value | β (95% CI) p-value | OR (95% CI) p-value |
| **Model 1**^b^, n=26,693 |  |  |  |  |
| Floor area, square feet (per IQR) | -0.238 (-0.399,-0.077) 0.004 | -0.330 (-0.577,-0.082) 0.009 | -0.268 (-0.445,-0.091) 0.003 | 0.945 (0.905,0.987) 0.010 |
| Housing units per block (per IQR) | 0.511 (0.2309,0.792) <0.001 | 0.808 (0.355,1.260) <0.001 | 0.610 (0.293,0.927) <0.001 | 1.094 (1.022,1.170) 0.009 |
| Neighbourhood residential density, units/Km^2^ (0.5 mi, per IQR) | -0.242 (-0.405,-0.080) 0.004 | -0.327 (-0.584,-0.070) 0.013 | -0.271 (-0.452,-0.089) 0.004 | 0.946 (0.908,0.985) 0.008 |
| **Model 2**^c^, n=16,947 |  |  |  |  |
| Floor area, square feet (per IQR) | -0.413 (-0.608,-0.220) <0.001 | -0.508 (-0.810,-0.206) 0.001 | -0.445 (-0.654,-0.237) <0.001 | 0.930 (0.878,0.985) 0.013 |
| Housing units per block (per IQR) | 0.348 (0.012,0.684)  0.042 | 0.413 (-0.129,0.955) 0.135 | 0.370 (-0.006,0.745) 0.054 | 1.068 (0.976,1.168) 0.153 |
| Neighbourhood residential density, units/Km^2^ (0.5 mi, per IQR) | 0.067 (-0.128,0.263) 0.499 | -0.056 (-0.368,0.256) 0.726 | 0.026 (-0.192,0.245) 0.814 | 1.008 (0.956,1.063) 0.779 |
| **Model 3**^d^, n=11,663 |  |  |  |  |
| Floor area, square feet (per IQR) | -0.319 (-0.532,-0.106) 0.003 | -0.363 (-0.660,-0.066) 0.017 | -0.334 (-0.554,-0.113) 0.003 | 0.904 (0.827,0.989) 0.028 |
| Housing units per block (per IQR) | 0.229 (-0.131,0.589) 0.213 | 0.416 (-0.126,0.957) 0.132 | 0.291 (-0.097,0.679) 0.141 | 1.026 (0.890,1.182) 0.723 |
| Neighbourhood residential density, units/Km^2^ (0.5 mi, per IQR) | 0.073 (-0.134,0.280) 0.488 | -0.122 (-0.441,0.198) 0.455 | 0.008 (-0.219,0.235) 0.943 | 0.958 (0.880,1.044) 0.327 |

^a^Model with neighbourhood environment (residential density, density of public transport and terrain) measured within 0.5-mile (805m) street catchment of geocoded participants’ residence.

^b^Model 1represents fully-adjusted cross-sectional model at baseline accounting for socio-demographics (age, sex, marital status, employment status, educational attainment, household income), lifestyle (smoking status, alcohol intake frequency, number of family members, shared living, housing type), comorbidities (obesity, cardiac heart disease) and environment (housing floor level, density of public transport and terrain).

^c^Model 2 represents fully-adjusted cross-sectional model at wave 2 accounting for socio-demographics (age, sex, marital status, employment status, educational attainment, household income), lifestyle (smoking status, alcohol intake frequency, number of family members, shared living, housing type), comorbidities (obesity, cardiac heart disease) and environment (housing floor level, density of public transport and terrain).

^d^Model 3 represents fully-adjusted longitudinal model (for participants who were followed-up and were non-hypertensive at baseline) accounting for socio-demographics (age, sex, marital status, employment status, educational attainment, household income), lifestyle (smoking status, alcohol intake frequency, number of family members, shared living, housing type), comorbidities (obesity, cardiac heart disease) and environment (housing floor level, density of public transport and terrain) and logarithm of follow-up time.

IQR: interquartile range, Km: Kilometre mi: mile, OR: odds ratio, β: beta, CI: confidence interval.

**Table K. Associations of housing environment exposures with blood pressure outcomes and hypertension among FAMILY Cohort participants aged 16 or above, with age as a continuous variable**

| **Housing environment^a^** | **Diastolic blood pressure (mm Hg)** | **Systolic blood pressure (mm Hg)** | **Mean arterial pressure (mm Hg)** | **Incident hypertension*** |
| --- | --- | --- | --- | --- |
|  | β (95% CI) p-value | β (95% CI) p-value | β (95% CI) p-value | OR (95% CI) p-value |
| **Model 1**^b^, n=30,439 |  |  |  |  |
| Floor area, square feet (per IQR) | -0.228 (-0.378,-0.078) 0.003 | -0.291 (-0.523,-0.059) 0.014 | -0.249 (-0.414,-0.084) 0.003 | 0.961 (0.924,0.9996) 0.048 |
| Housing units per block (per IQR) | 0.526 (0.260,0.793) <0.001 | 0.844 (0.419,1.268) <0.001 | 0.632 (0.334,0.931) <0.001 | 1.109 (1.040,1.182) 0.002 |
| Neighbourhood residential density, units/Km^2^ (0.5 mi, per IQR) | -0.283 (-0.436,-0.131) <0.001 | -0.366 (-0.608,-0.125) 0.003 | -0.311 (-0.482,-0.141) <0.001 | 0.940 (0.905,0.976) 0.001 |
| **Model 2**^c^, n=20,244 |  |  |  |  |
| Floor area, square feet (per IQR) | -0.264 (-0.442,-0.086) 0.004 | -0.421 (-0.689,-0.153) 0.002 | -0.317 (-0.506,-0.127) 0.001 | 0.937 (0.891,0.986) 0.013 |
| Housing units per block (per IQR) | 0.452 (0.146,0.758) 0.004 | 0.619 (0.133,1.104) 0.012 | 0.508 (0.168,0.847) 0.003 | 1.074 (0.990,1.166) 0.087 |
| Neighbourhood residential density, units/Km^2^ (0.5 mi, per IQR) | 0.036 (-0.142,0.215) 0.689 | -0.068 (-0.350,0.214) 0.636 | 0.002 (-0.197,0.200) 0.988 | 1.019 (0.970,1.071) 0.453 |
| **Model 3**^d^, n=13,895 |  |  |  |  |
| Floor area, square feet (per IQR) | -0.207 (-0.400,-0.013) 0.037 | -0.267 (-0.538,0.005) 0.054 | -0.227 (-0.427,-0.026) 0.027 | 0.916 (0.844,0.995) 0.037 |
| Housing units per block (per IQR) | 0.366 (0.038,0.693) 0.028 | 0.598 (0.107,1.088) 0.017 | 0.443 (0.092,0.794) 0.013 | 1.034 (0.909,1.175) 0.614 |
| Neighbourhood residential density, units/Km^2^ (0.5 mi, per IQR) | 0.071 (-0.118,0.261) 0.460 | -0.116 (-0.406,0.174) 0.434 | 0.009 (-0.197,0.216) 0.931 | 0.986 (0.911,1.068) 0.735 |

^a^Model with neighbourhood environment (residential density, density of public transport and terrain) measured within 0.5-mile (805m) street catchment of geocoded participants’ residence.

^b^Model 1represents fully-adjusted cross-sectional model at baseline accounting for socio-demographics (age as a continuous variable, sex, marital status, employment status, educational attainment, household income), lifestyle (smoking status, alcohol intake frequency, number of family members, shared living, housing type), comorbidities (obesity, cardiac heart disease) and environment (housing floor level, density of public transport and terrain).

^c^Model 2 represents fully-adjusted cross-sectional model at wave 2 accounting for socio-demographics (age as a continuous variable, sex, marital status, employment status, educational attainment, household income), lifestyle (smoking status, alcohol intake frequency, number of family members, shared living, housing type), comorbidities (obesity, cardiac heart disease) and environment (housing floor level, density of public transport and terrain).

^d^Model 3 represents fully-adjusted longitudinal model (for participants who were followed-up and were non-hypertensive at baseline) accounting for socio-demographics (age as a continuous variable, sex, marital status, employment status, educational attainment, household income), lifestyle (smoking status, alcohol intake frequency, number of family members, shared living, housing type), comorbidities (obesity, cardiac heart disease) and environment (housing floor level, density of public transport and terrain) and logarithm of follow-up time.

IQR: interquartile range, Km: Kilometre mi: mile, OR: odds ratio, β: beta, CI: confidence interval..

**Table L: Association of liveable floor area and housing units per block with blood pressure outcomes by population sub-groups of sex, age, income categories, employment status and housing types for n=20,244 participants at wave 2.**

|  | **n** | **Diastolic blood pressure**  **(mm Hg)**  β (95% CI) p-value | **Systolic blood pressure**  **(mm Hg)**  β (95% CI) p-value | **Mean arterial pressure**  **(mm Hg)**  β (95% CI) p-value |
| --- | --- | --- | --- | --- |
|  |  |  |  |  |
| **Liveable floor area in square feet (per IQR increment)** | | | | |
| **Sex** |  |  |  |  |
| Male | 9,116 | -0.255 (-0.524,0.013) 0.063 | -0.384 (-0.772,0.003) 0.052 | -0.298 (-0.579,-0.017) 0.037 |
| Female | 11,128 | -0.377 (-0.607,-0.146) 0.001 | -0.526 (-0.897,-0.154) 0.006 | -0.426 (-0.677,-0.176) 0.001 |
| p-interaction |  | 0.041 | 0.045 | 0.026 |
| **Age groups** |  |  |  |  |
| ≤40 | 6,886 | -0.183 (-0.464,0.097) 0.200 | -0.235 (-0.583,0.113) 0.185 | -0.201 (-0.477,0.076) 0.154 |
| >40-≤50 | 4,489 | -0.403 (-0.804,-0.001) 0.049 | -0.578 (-1.114,-0.042) 0.035 | -0.461 (-0.882,-0.040) 0.032 |
| >50-≤60 | 4,130 | -0.503 (-0.911,-0.094) 0.016 | -0.616 (-1.227,-0.005) 0.048 | -0.541 (-0.995,-0.086) 0.020 |
| >60 | 4,739 | -0.352 (-0.702,-0.003) 0.048 | -0.447 (-1.103,0.209) 0.182 | -0.384 (-0.775,-0.007) 0.054 |
| p-interaction |  | 0.943 | 0.351 | 0.779 |
| **Income categories (HK$)** |  |  |  |  |
| Low (<4,999) | 8,039 | -0.422 (-0.699,-0.145) 0.003 | -0.591 (-1.074,-0.108) 0.017 | -0.478 (-0.780,-0.177) 0.002 |
| Low-med ($5,000-9,999) | 3,736 | -0.255 (-0.743,0.234) 0.307 | -0.407 (-1.166,0.352) 0.293 | -0.305 (-0.846,0.235) 0.268 |
| High-med (10,000-14,999) | 3,448 | -0.378 (-0.873,0.117) 0.134 | 0.097 (-0.678,0.871) 0.807 | -0.220 (-0.768,0.328) 0.431 |
| High (≥$15,000) | 5,021 | -0.225 (-0.522,0.072) 0.138 | -0.424 (-0.827,-0.021) 0.039 | -0.291 (-0.603,0.020) 0.067 |
| p-interaction |  | 0.748 | 0.345 | 0.695 |
| **Employment status** |  |  |  |  |
| Employed | 10,773 | -0.308 (-0.566,-0.050) 0.019 | -0.437 (-0.791,-0.083) 0.016 | 0.351 (-0.623, -0.079) 0.011 |
| Homemaker/student/others | 6,358 | -0.521 (-0.836,-0.207) 0.001 | -0.715 (-1.206,-0.224) 0.004 | -0.586 (-0.929, -0.243) 0.001 |
| Retiree/unemployed | 3,113 | -0.029 (-0.411, 0.353) 0.881 | -0.001 (-0.676,0.674) 0.997 | -0.020 (-0.423, 0.384) 0.923 |
| p-interaction |  | 0.066 | 0.376 | 0.104 |
| **Housing type** |  |  |  |  |
| Public | 9,495 | -0.901 (-1.349,-0.453) <0.001 | -1.035 (-1.735,-0.335) 0.004 | -0.945 (-1.440,-0.450) <0.001 |
| Private/subsidized | 10,749 | -0.215 (-0.407,-0.023) 0.029 | -0.334 (-0.630,-0.037) 0.028 | -0.254 (-0.459,-0.050) 0.015 |
| p-interaction |  | 0.002 | 0.073 | 0.006 |
| **Housing units per block (per IQR increment)** | | | | |
| **Sex** |  |  |  |  |
| Male | 9,116 | 0.438 (-0.019,0.895) 0.060 | 0.694 (-0.022,1.411) 0.057 | 0.523 (0.022,1.025) 0.041 |
| Female | 11,128 | 0.402 (-0.005,0.809) 0.053 | 0.545 (-0.111,1.200) 0.103 | 0.450 (-0.007,0.906) 0.054 |
| p-interaction |  | 0.051 | 0.098 | 0.050 |
| **Age groups** |  |  |  |  |
| ≤40 | 6,886 | 0.489 (0.019,0.959) 0.042 | 0.697 (0.091,1.302) 0.024 | 0.558 (0.082,1.034) 0.021 |
| >40-≤50 | 4,489 | 0.455 (-0.287,1.196) 0.229 | 0.466 (-0.604,1.536) 0.393 | 0.458 (-0.355,1.272) 0.269 |
| >50-≤60 | 4,130 | 0.280 (-0.428,0.988) 0.437 | 0.468 (-0.663,1.599) 0.417 | 0.343 (-0.456,1.142) 0.400 |
| >60 | 4,739 | 0.398 (-0.198,0.995) 0.191 | 0.733 (-0.385,1.851) 0.199 | 0.510 (-0.195,1.215) 0.156 |
| p-interaction |  | 0.768 | 0.838 | 0.859 |
| **Income categories (HK$)** |  |  |  |  |
| Low (<4,999) | 8,039 | 0.335 (-0.132,0.802) 0.160 | 0.533 (-0.249,1.314) 0.182 | 0.401 (-0.125,0.927) 0.135 |
| Low-med ($5,000-9,999) | 3,736 | 0.279 (-0.383,0.941) 0.409 | 0.462 (-0.617,1.542) 0.401 | 0.340 (-0.414,1.094) 0.376 |
| High-med (10,000-14,999) | 3,448 | 0.398 (-0.303,1.099) 0.266 | 0.547 (-0.532,1.625) 0.320 | 0.448 (-0.320,1.215) 0.253 |
| High (≥$15,000) | 5,021 | 0.727 (-0.024,1.478) 0.058 | 0.753 (-0.274,1.780) 0.151 | 0.736 (-0.06,1.532) 0.070 |
| p-interaction |  | 0.732 | 0.752 | 0.746 |
| **Employment status** |  |  |  |  |
| Employed | 10,773 | 0.425 (-0.007,0.857) 0.054 | 0.543 (-0.100,1.185) 0.098 | 0.464 (-0.008, 0.936) 0.054 |
| Homemaker/student/others | 6,358 | 0.358 (-0.151,0.867) 0.168 | 0.643 (-0.179,1.464) 0.125 | 0.453 (-0.113, 1.019) 0.117 |
| Retiree/unemployed | 3,113 | 0.520 (-0.257, 1.298) 0.190 | 0.800 (-0.607,2.207) 0.265 | 0.614 (-0.290,1.517) 0.183 |
| p-interaction |  | 0.747 | 0.491 | 0.629 |
| **Housing type** |  |  |  |  |
| Public | 9,495 | 0.145 (-0.257,0.548) 0.478 | 0.262 (-0.391,0.914) 0.432 | 0.184 (-0.266, 0.634) 0.423 |
| Private/subsidized | 10,749 | 0.618 (0.112, 1.123) 0.017 | 0.963 (0.162,1.763) 0.018 | 0.733 (0.170,1.295) 0.011 |
| p-interaction |  | 0.373 | 0.455 | 0.373 |

^a^Fully-adjusted models accounting for socio-demographics (age, sex, marital status, employment status, educational attainment, income), lifestyle (smoking status, alcohol intake frequency, number of family members, shared living, housing type), comorbidities (obesity, cardiac heart disease) and environment (housing floor level density of public transport and terrain within 0.5-mile catchment).

β: beta, CI: confidence interval.
