# Supplementary material for: Liveable residential space, residential density, and hypertension in Hong Kong: A population-based cohort study
Source: PLoS Med. 2021 Nov 2;18(11):e1003824. doi: 10.1371/journal.pmed.1003824 (PMC8562807; doi:10.1371/journal.pmed.1003824)
Supplement: S3 Appendix — Fig A: An illustration showing the attributes of housing exposures in the developed HKHED database; livable floor area, building units per block and neighborhood residential density. km: kilometer. Fig B: Flowchart of the selection of participants for cross-sectional analyses at baseline and wave 2. Fig C: Flowchart of the selection of participants for longitudinal analyses on linked data across 2 waves. Fig D: Density plot showing the distribution of propensity scores of the incident hypertension model in the control group (participants who did not change their residential address between the 2 waves) marked as 0, and the treatment group (participants who changed residence to lower liveable floor area) marked as 1 after matching. HKHED, Hong Kong Housing Environment Database. (DOCX) [file pmed.1003824.s003.docx]

**Liveable residential space, residential density and hypertension in Hong Kong: A population-based cohort study**

Chinmoy Sarkar^1,2^†*, Ka Yan Lai^1^†, Michael Y. Ni^1,2,3^, Sarika Kumari^1^, Gabriel M. Leung^2^, Chris Webster^1^

^1^*Healthy High Density Cities Lab, HKUrbanLab, The University of Hong Kong, Knowles Building, Pokfulam Road, Pokfulam, Hong Kong Special Administrative Region, China.*

*^2^School of Public Health, The University of Hong Kong, Patrick Manson Building, Sassoon Road, Pokfulam, Hong Kong Special Administrative Region, China.*

*^3^The State Key Laboratory of Brain and Cognitive Sciences, The University of Hong Kong, Hong Kong Special Administrative Region, China.*

*†These authors share first authorship on this work.*

**Corresponding author*

**Supporting information:**

S3 Appendix: Supporting Figures


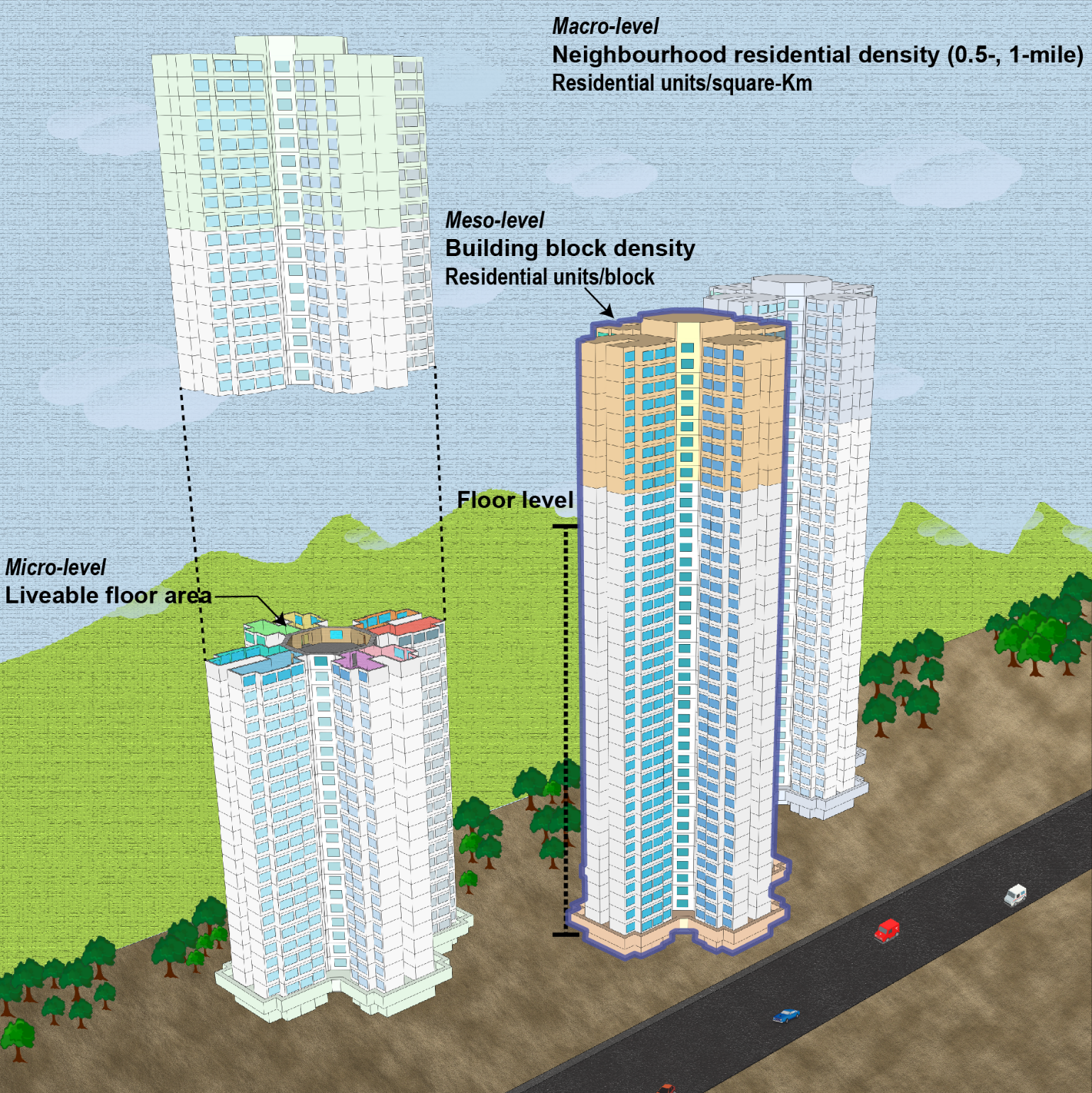
.

**Fig A. An illustration showing the attributes of housing exposures in the developed HKHED database; livable floor area, building units per block and neighbourhood residential density.**

Km: kilometre.

Missing data on outcomes; **n=1,795**

(DBP=990; SBP=878; MAP=1,041; Hypertension=1,795)

Missing data on residential exposures (participants’ address error/couldn’t be geocoded); **n=2,307**

**Baseline**

Participants aged≥16

**n=41,758**

**Target sample** with outcome & exposure data

**n=37,656**

Missing socio-demographics; **n=6,326**

(marital status=54; educational qualification=182; employment status=3,649; income=2,810)

Participants aged≥16 with outcome data **n=39,963**

Missing data on lifestyle and risk factors; **n=891**

(housing type=1; smoking status=40; alcohol intake frequency=241; BMI status=706; coronary heart disease=256)

**Sample with complete data** used in primary analysis

**n=30,439**

Missing data on outcomes; **n=1,370**

(DBP=618; SBP=664; MAP=903; Hypertension=1,370)

Missing data on residential exposures (participants’ address error/couldn’t be geocoded); **n=1,497**

**Wave 2**

Participants aged≥16

**n=28,076**

**Target sample** with outcome & exposure data

**n=25,209**

Missing socio-demographics; **n=3,577**

(marital status=34; educational qualification=37; employment status=2,599; income=1,114)

Participants aged≥16 with outcome data **n=26,706**

Missing data on lifestyle and risk factors; **n=1,388**

(housing type=753; smoking status=35; alcohol intake frequency=48; BMI status=788; coronary heart disease=144)

**Sample with complete data** used in primary analysis

**n=20,244**

**Fig B. Flowchart of the selection of participants for cross sectional analyses at baseline and wave 2.**

**Wave 2 Target sample**

**n=25,209**

Exclusions; **n=8,821**

- New recruits at Wave 2 (no baseline data) =1,418
- Missing data on outcomes/ exposure at baseline =1,461
- Hypertensive at baseline=5,942

**Linked Baseline** and **Wave 2** data

**n=16,388**

**Linked sample with complete data** used in primary analysis

**n=13,895**

Missing covariates at Wave 2; **n=2,493**

- Missing data on socio-demographics=2,143
- Missing data on lifestyle and risk factors=350

**Fig C. Flowchart of the selection of participants for longitudinal analyses on linked data across two waves.**


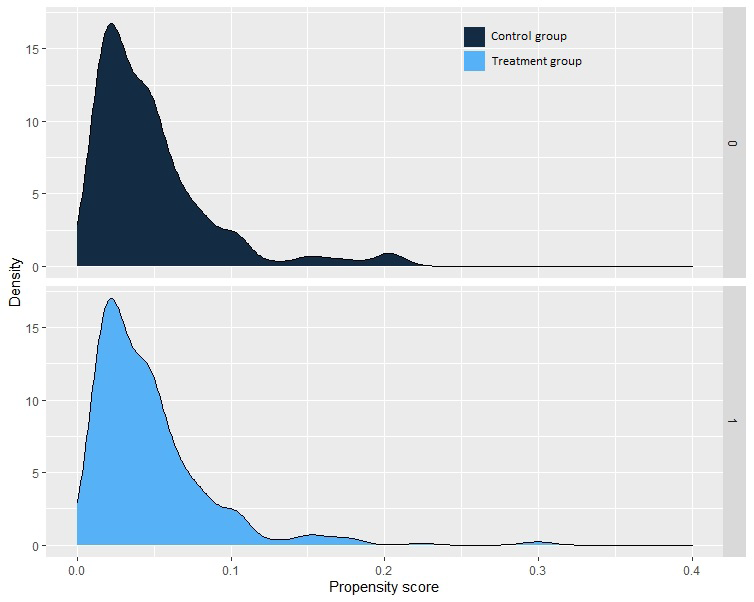


**Fig D. Density plot showing the distribution of propensity scores of the incident hypertension model in the control group (participants who did not change their residential address between the two waves) marked as 0, and the treatment group (participants who changed residence to lower liveable floor area) marked as 1 after matching.**
